# Neutrophil extracellular traps in homeostasis and disease

**DOI:** 10.1038/s41392-024-01933-x

**Published:** 2024-09-20

**Authors:** Han Wang, Susan J. Kim, Yu Lei, Shuhui Wang, Hui Wang, Hai Huang, Hongji Zhang, Allan Tsung

**Affiliations:** 1 Department of Gastroenterology, Tongji Hospital, Tongji Medical College, Huazhong University of Science and Technology, Wuhan, Hubei China; 2 Department of Surgery, School of Medicine, University of Virginia, Charlottesville, VA USA; 3 Department of Medical Genetics, School of Basic Medicine, Tongji Medical College, Huazhong University of Science and Technology, Wuhan, Hubei China; 4 Feinstein Institutes for Medical Research, Manhasset, NY USA

**Keywords:** Innate immunity, Tumour immunology, Adaptive immunity

## Abstract

Neutrophil extracellular traps (NETs), crucial in immune defense mechanisms, are renowned for their propensity to expel decondensed chromatin embedded with inflammatory proteins. Our comprehension of NETs in pathogen clearance, immune regulation and disease pathogenesis, has grown significantly in recent years. NETs are not only pivotal in the context of infections but also exhibit significant involvement in sterile inflammation. Evidence suggests that excessive accumulation of NETs can result in vessel occlusion, tissue damage, and prolonged inflammatory responses, thereby contributing to the progression and exacerbation of various pathological states. Nevertheless, NETs exhibit dual functionalities in certain pathological contexts. While NETs may act as autoantigens, aggregated NET complexes can function as inflammatory mediators by degrading proinflammatory cytokines and chemokines. The delineation of molecules and signaling pathways governing NET formation aids in refining our appreciation of NETs’ role in immune homeostasis, inflammation, autoimmune diseases, metabolic dysregulation, and cancer. In this comprehensive review, we delve into the multifaceted roles of NETs in both homeostasis and disease, whilst discussing their potential as therapeutic targets. Our aim is to enhance the understanding of the intricate functions of NETs across the spectrum from physiology to pathology.

## Introduction

Neutrophils are the first line of defense within the innate immune system, crucial for protecting the host against pathogens. Alongside traditional defense mechanisms, recent attention has focused on unique fibrous web-like chromatin structures, termed neutrophil extracellular traps (NETs).^1,2^ NETs aid neutrophils in immobilizing and trapping pathogens, thereby contributing to host defense.^3–5^ This process relies on associated histones, proteolytic enzymes from granules, and enzymatic myeloperoxidase (MPO).^1,2^ Accumulating evidence strongly supports the direct and indirect regulatory effects of NETs on both adaptive and innate immunity,^6–8^ playing a crucial role in immune homeostasis. Moreover, NETs contribute specific mechanisms to potentiate immunothrombosis,^9–12^ potentially playing a protective role in the context of infection.^13^

NETs are typically formed and exhibit antibacterial activity in a variety of infectious conditions, including bacterial, parasitic, and fungal infections,^14,15^ where these pathogens can act as stimuli to induce NET formation. Impaired NET function may facilitate pathogen evasion from the immune system and create a niche for chronic infection.^16–18^ Nevertheless, akin to a double-edged sword, sustained inflammation or persistent stimuli can lead to excessive NET formation, thereby exacerbating tissue damage during inappropriate inflammation. Additionally, NET formation is observed in nonpathogenic conditions, including but not limited to sterile inflammation, autoimmune disorders, metabolic dysregulation, vasculitis, thrombosis, and carcinogenesis when dysregulated.^19–21^ Under sterile conditions, NETs can be induced by interleukin-8 (IL-8),^22^ immune complexes,^23^ crystals,^24^ or damage-associated molecular patterns (DAMPs), such as high mobility group Box 1 (HMGB1).^25^ Evidence thus far suggests that NETs play dual roles in these nonpathogenic conditions. On one hand, NETs may act as autoantigens in autoimmune conditions, contributing to tissue destruction, amplifying the inflammatory cascade, and promoting thrombosis formation.^19–21^ On the other hand, aggregated NETs formed during sterile inflammation, containing a diverse array of enzymes, have the potential to serve as inflammatory mediators by degrading proinflammatory cytokines and chemokines, thereby promoting inflammation resolution and wound healing.^10,11^ Despite the controversial role of NETs, major studies confirm their more detrimental roles in nonpathogenic inflammation.

Emerging evidence emphasizes the protumorigenic role of NETs in various cancers,^26–28^ primarily due to their contribution to cell damage and regeneration, leading to subsequent excessive inflammation. NETs have been reported to promote tumor cell proliferation,^29^ metastasis,^30–32^ immunosuppression,^33,34^ and cancer-associated thrombosis.^35^ Additionally, NETs can capture circulating tumor cells and facilitate their colonization.^36^ The antitumor effects of NETs vary depending on tumor type and microenvironment.^37^ While the debate continues regarding whether NETs inhibit or promote tumor progression, their role in promoting tumor development appears more evident.^38^ Accumulated NETs provide an immunosuppressive microenvironment favoring the survival of premalignant cells and cancer cells.^39^ Elevated NET markers correlate with poor clinical outcomes in cancer patients and may serve as prognostic indicators.^40–42^ This review explores the molecular mechanisms underlying NET formation and clearance, along with recent advances in comprehending how NETs contribute to both infection defense and pathologies associated with various diseases, including specific inflammatory, autoimmune, thrombotic, and cancerous conditions. Additionally, we provide an overview of current clinical trials and therapies targeting NETs, offering insights into the development of therapeutic strategies targeting NETs in the clinical practice.

## History of research on NETs

NETs have a rich history in research, beginning approximately two decades ago. NETs were first described in the early 2000s as a protective mechanism against pathogenic bacteria,^1^ which was subsequently expanded to protection against yeast^43^ and protozoal species. Quickly thereafter, NETs were associated with a variety of human disease processes, first described in the female reproductive tract.^44–46^ As NETs continued to be studied, it was revealed that certain bacteria expressed endonucleases that degraded NETs as a protective mechanism.^47–49^ As these mechanisms for pathogen evasion^50,51^ became better understood, this led to research developments on harnessing exogenous methods of inhibition or degradation to address human pathology.

In 2007, models of NET activity began to expand into other animal models including fish,^52^ and zebrafish,^53^ demonstrating the conserved function of NETs across species. Simultaneously, research shifted toward elucidating the mechanism of NETosis, as well as the structural components that are responsible for their functionality; Pentraxin-3 (PTX3) was identified as a structural protein dotted on NETs^54^ and the connection with toll-like receptor-mediated activation, which was monumental in the study of NETs in sepsis.

Thus began the era of NETs as prognostic biomarkers in the clinical setting,^55–57^ particularly in the realm of autoimmune disease. Beginning in 2010, the role of NETs in cancer began to emerge,^58^ first being implicated in non-human animal models. In 2011, exogenous deoxyribonuclease (DNase) came to the forefront as a modality of NET degradation in human disease models^59^ and has remained a primary agent for NET degradation in current pre-clinical and clinical trials. Causative mechanisms for how their degradation led to these improved outcomes expanded substantially,^60,61^ leading to studies that focused on inhibiting NET formation^62,63^ in addition to the degradation that was emphasized previously.

Quickly after the association between human cancers and NETs was made, it became evident that NETs were also responsible for malignancy-related complications such as tumor-associated thrombosis^64,65^ and metastases.^66^ Due to the immunogenic environment of cancers, it was natural that at this time the ability of NETs to modulate the innate as well as the adaptive immune microenvironment was also recognized, notably in terms of modulating the T cell compartment.^67^

The first human observational studies regarding NETs in critical care literature was published in 2014,^68^ then rapidly expanded to the transplant^69^ and cardiac^70,71^ populations. With these observational studies, the in-vivo effects of NETs became better understood^72^ and the use of NET components in prediction models grew.^73–76^ Furthermore, the beginnings of high throughput biomarker detection systems started to be explored.^77,78^

Beginning in 2016, the concept of iatrogenic NET induction was introduced, with commonly used medical tools such as antibiotics^79,80^ and ventilators^81^ implicated in NET formation and subsequent poor outcomes. A key cause of iatrogenic NET induction was found to be chemotherapy, leading to treatment resistance.^82^ In addition to chemotherapy resistance, significant advances were made in identifying the role of NETs in metastatic disease, with a heavy emphasis in their role in modulating the immune microenvironment,^83–85^ inducing escape mechanisms such as epithelial-mesenchymal transition (EMT),^33,86,87^ and migration.^88–91^ This ultimately led to the expansion of research on NET targeting therapies,^92–96^ and mitigating the adverse effects of NETs. In the 2020s, agents targeting NET degradation or inhibition have been expanded outside of DNase, exploring thrombomodulin^97^ or necrostatin-1^98^ as promising agents in the preclinical space. Furthermore, more selective targeting of NET components has become more prominent, demonstrating similar outcome efficacy as degradation.^99^ Interestingly, the role of exercise in reversing the effects of NETs has become a popular topic of research interest^100,101^ in recent years.

**Fig. 1:**
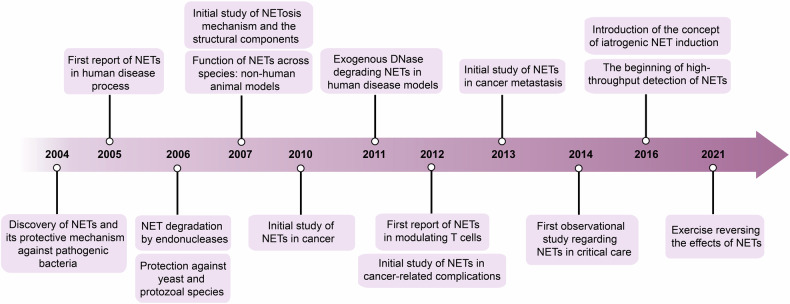
History of research on NETs. The major discoveries related to NETs, from their initial identification and role in pathogen eradication to their involvement in diseases such as cancer. It illustrates the progression of research over time and the increasing recognition of their clinical significance. This figure was created by Adobe Illustrator Artwork 16.0 (Adobe Systems, USA)

While the connection of NETs and the immune system, particularly in its modulation of other immune players^102^ has been well researched in the decades of NET-related research, NETs have also been found to connect to a myriad of homeostatic mechanisms, in particular cellular metabolism.^103,104^ As the knowledge of NETs multi-functionality and its role in disease has expanded in recent years, research has shifted to elucidating its role as a prognostic and predictive biomarker in acute stages of disease,^105–107^ and strides have been taken to elucidate its role in other disease processes through genomics research^108^ within the past five years. Research thus far has illustrated the wide breadth and comprehensive scope of NET functionality and continues to make rapid advancements (Fig. 1).

## Structure of NETs

NETs are web-like extracellular filamentous structures released by activated neutrophils. A distinctive feature of NETs is the exposed DNA fibers with diameters of 15-17 nm formed by decondensed neutrophil nuclear chromatin, which are important components of NETs. Although DNA is extruded from NETs for defense purposes, it has both antimicrobial and pro-inflammatory properties throughout the immune responses.^109^ High concentrations of DNA can chelate divalent metal cations, which can destroy the membranes of bacteria. As a cue for tissue damage locally or programmed cell death, extracellular DNA can be rapidly degraded by circulating nucleases, as well as engulfed by phagocytes.^110,111^ Impairment of the process might trigger a strong inflammatory response. Mitochondrial DNA (mtDNA) is another source of NETs and acts as a DAMP capable of triggering a pro-inflammatory response. The rapid activation of important NETs by mtDNA stimulates other neutrophils, which amplify the inflammatory responses by further releasing NETs through a positive feedback mechanism.^112,113^

Notably, histones, including H1, H2A, H2B, H3, and H4, are also major components of NETs, accounting for ~70% of the proteins of NETs.^114^ Although unstimulated neutrophils have the same proportion of all core histones, there are higher amounts of H2A and H2B compared with H3 and H4 in NETing neutrophils.^115^ Posttranslational modifications of histones also have been found in NETs, even during NET formation. As serine proteases shear the histones of NETs during NET formation, histones of NETs are 2–5 kDa smaller than those unstimulated.^116^ Acetylation is another modification neutralizing the positive charges in histones, allowing them to detach from DNA and chromatin loss.^117^ The conversion of arginine into citrulline by peptidyl arginine deiminases (PAD) is named histone citrullination, and it is noteworthy that citrullinated histones have been recognized as one of the major sources of autoantibodies in certain autoimmune diseases, such as rheumatoid arthritis (RA).^118,119^ In addition, histones also have immunophysiological characteristics, such as antimicrobial activity, cytotoxicity, and immunomodulation. Extracellular histones can cause potent pro-inflammatory responses, leading to organ damage and even death.^111^

Furthermore, cytoplasmic proteins (including S100 calcium-binding proteins A8/A9/A12) and granular proteins (such as MPO), neutrophil elastase (NE), proteinase 3 (PRTN3), cathepsin G, neutrophil defensins) bind in globular patterns to NETs. During the formation and release of NETs, the chromatin swells up, allowing the granule components and cellular components to come into contact.^111,120^ The toxicity of the various components released by degranulation might cause tissue damage at the site of infection and play an important role in some non-infectious diseases, especially autoimmune diseases and tumors.

## Mechanisms of NET formation

### Activation of NETs

NETs catch a wide range of bacterial pathogens and prevent their spread. Previous studies have shown that *Streptococcus suis* (*S. suis*) can be recognized by toll-like receptors (TLRs), which activate NET formation in an nicotinamide adenine dinucleotide phosphate oxidase (NOX)-dependent manner.^121^ Although small pathogens exhibit weaker stimulatory effects of NETs, small bacteria have been reported to induce NET formation. This occurs when small microorganisms evade death by phagosomes and tend to aggregate. The size of the external invaders is not a determining factor in activating formation of NETs, but the number of particles in the neutrophil cytoplasm may be a sensitive indicator, as *Staphylococcus aureus* (*S. aureus*) aggregates when exposed to plasma in a murine model of sepsis, which triggers NET formation.^122,123^ Moreover, NET activation has been perceived in response to virus infection caused by respiratory syncytial virus (RSV), human immunodeficiency virus (HIV), hepatitis B virus (HBV), and severe acute respiratory syndrome coronavirus 2 (SARS-CoV-2).^124–127^ In RSV and HIV-induced infections, NETs seem to be beneficial to the immune systems, whereas NET formation in patients with Coronavirus disease 2019 (COVID-19) has been shown to be deleterious.

In addition to pathogens, different immunological stimuli (including interleukins, interferons, autoantibodies, and immune complexes), tumor-associated stimuli (including granulocyte-colony stimulating factor (G-CSF), C-X-C motif chemokine ligands (CXCLs)), lipopolysaccharides (LPS) and DAMPs can also promote the formation of NETs. The stimuli may activate the cell surface receptors of neutrophils; for example, immune complexes activate the FcgRIIIb receptor, CXCLs recognize CXC chemokine receptors (CXCRs), C3a recognizes C3a receptor (C3aR), as well as HMGB1 recognizes receptor of advanced glycation end products (RAGE) and TLR4.^2,128,129^ Upon activation of receptors on neutrophils by stimuli, a variety of intracellular signaling mechanisms are further activated, resulting in NET formation. Notably, activated platelets and endothelial cells, the important parts of microenvironment in vivo, have also been reported to exhibit a role in activating NET formation in diseases such as sepsis, stroke and tumors.^130,131^

Phorbol 12-myristate 13-acetate (PMA) is a well-known activator of NET formation used for scientific studies. Recent studies have demonstrated that certain metabolites and external environmental factors, and also induce NET activation. Metabolites from gut microbiome dysbiosis and free fatty acids are involved in both infectious and non-infectious diseases by promoting NET formation.^132^ Cigarette smoke and PM2.5 might contribute to pulmonary diseases through activating NETs as well.^133,134^ Moreover, bleomycin has been shown to induce NET formation and fibrosis in the lungs of mice.^135^ Diverse particles also have been shown to induce NET formation, such as hydrophobic nanoparticles, acicular microparticles, and other natural and artificial crystals. Nanoparticles with specific surface properties can be used as adjuvants that stimulate NETs.^136^ Munoz et al. found that lysosomal destabilization and nuclear disassembly occur simultaneously after exposure of neutrophils to nonpolar nanoparticles, followed by the formation of nicotinamide adenine dinucleotide phosphate (NADPH) oxidase-dependent chromatin externalization, suggesting that, in addition to exogenous factors, lysosomal leakage in neutrophils might also trigger NET formation.^137^ However, to date, the formation of NETs in response to a variety of stimuli is not fully understood.

### NET formation pathways

In various diseases, neutrophils are recruited into the microenvironment by diverse mediators to form NETs. Chemokine concentration gradients influence the direction of neutrophil migration. For instance, local tissue injury can lead to increased production of G-CSF, which stimulates neutrophil recruitment.^138^ Additionally, CXCLs and C-C motif chemokine ligands (CCLs), such as CXCL1, CXCL5, and CCL2, play key roles in neutrophil recruitment in diseases.^139,140^

**Fig. 2:**
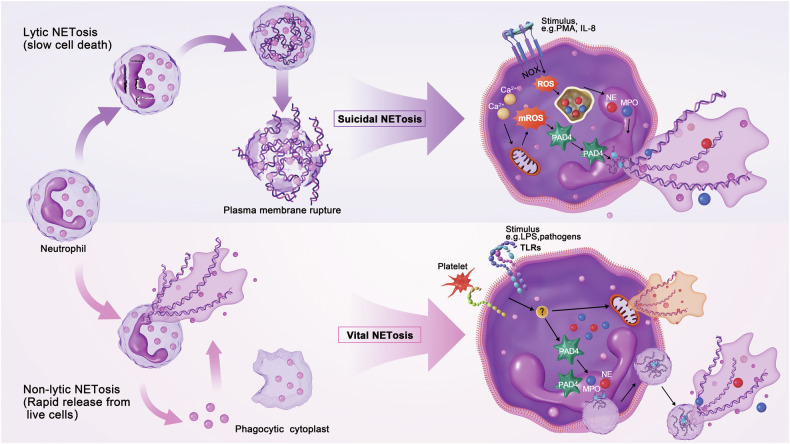
NET formation pathways. NET formation can be categorized into two main pathways. The first type is the classic pathway known as NETosis, which initiates with nuclear lobulation, followed by disassembly of nuclear membranes, loss of cellular polarization, chromatin decondensation, and eventual rupture of plasma membranes. An alternative pathway is termed non-lytic NETosis which can occur without cell death, where chromatin expulsion is accompanied by the release of granular proteins. These components are formed extracellularly, leaving behind active anucleate phagocytes with capabilities for microbial phagocytosis and chemotaxis. This figure was created by Adobe Photoshop CS6 (Adobe Systems, USA)

Although the specific process of NET formation differs depending on the stimuli, it can be categorized as two main pathways (Fig. 2). The first is a cell death pathway termed NETosis, which begins with nuclear delobulation, disassembly of nuclear membranes, a constant loss of cellular polarization, decondensation of chromatin, and eventually rupture of plasma membranes. This process of lytic cell death is that taking 2–4 h usually.^20,141^ An alternative pathway is non-lytic NETosis that can occur without cell death, whereby chromatin expulsion is accompanied by granular proteins release. These components are formed extracellularly, leaving behind active anucleate phagocytes with microbial phagocytosis and chemotaxis capabilities. This pathway occurs relatively quickly, usually within 5–60 min, but depends on the inducer.^20,142^

#### The lytic NETosis

The lytic NETosis pathway is also known as “suicide NETosis”, as well as NOX-dependent NETosis. Antibodies, microorganisms, cholesterol, and PMA can induce the lytic NETosis.^143^ These stimuli trigger the activation of signaling pathway proteins, leading to increased cytosolic calcium levels and activation of NOX. Further downstream, oxidase converts molecular oxygen to create reactive oxidative species (ROS). NE is located in the granules of phagocytosis in the resting neutrophils, partly bound to MPO and attached to the granule membranes, with another part in the lumen. ROS induces the activation of NE, as well as its release into the cytoplasm from the MPO-containing azurosome complex. NE binds to F-actin and mediates degradation of actin filaments. NE then translocates to the nucleus and partially cleaves histones to promote chromatin decondensation. Hydrogen peroxide releases NE into the cytoplasm selectively, which depends on MPO. However, inhibition of the enzymatic activity of MPO only delays rather than prevents NETosis, most likely because of the role of MPO in activating the hydrolytic activity of NE on bulky protein substrates.^144^

The role of the MPO-NE pathway is supported by studies of neutrophils from diabetes patients with hereditary MPO deficiency at high risk of infection.^145^ Bellaaouaj et al. have reported that mice with NE deficiency are more susceptible to sepsis and death,^146^ and NE inhibition prevents NET formation and rescues mice from ischemia/reperfusion injury, infection, and tumor.^147–150^ Lacking the NADPH oxidase in the respiratory burst pathway can decrease the ability to kill microorganisms, leading to recurrent microbial infections. Similarly, neutrophil elastase gene (ELANE) mutation is one of the most common genetic mutations in neutropenic patients. ELANE-induced neutropenia is associated with dysfunction of the theisprotease enzyme rather than due to NE deficiency. Patients with heterozygous mutations in the ELANE gene might develop severe life-threatening congenital neutropenia, or cyclic neutropenia with mild to moderate clinical characteristics.^151^

Recent studies have shown another nuclear chromatin-binding protein implicated in NETosis is DEK. Both DEK depletion and treatment with DEK-targeted aptamers attenuate inflammation in vivo and greatly impair NET formation, while NETosis can be reversed by addition of exogenous recombinant DEK protein, suggesting that chromatin decondensation mediated by DEK binding is similar to MPO.^152,153^

Another factor involved in NETosis is PAD4, which decreases the positive charge of histones, as well as their electrostatic interactions with DNA. The formation of a catalytically active conformation of this enzyme requires five calcium ionophores, which are always employed in studies on exploring the role of PAD4 in NETosis.^144^ ROS also promotes PAD4 activation.^154^ Citrullination mediated by PAD4 can be triggered by hydrogen peroxide, which can be reduced by inhibiting NADPH oxidase, indicating an association between PAD4 and production of ROS. The results of experiments with PAD4 inhibitor-treated cell lines or with neutrophils from mice with PAD4 deficiency are difficult to interpret because of low NET yields.^135^ For example, PAD4 inhibition prevents NET formation activated by nicotine rather than cholesterol crystals.^24,155^ However, studies with a variety of NET markers have shown that inhibition of PAD4 suppresses NET release in murine models of sepsis and cancer. Moreover, recent studies demonstrate that blockade of citrullination inhibits the pro-inflammatory effects of histones and the formation of atherosclerotic plaques in mice, but not NETosis. In contrast, granule proteases in mouse neutrophils may be indispensable for NETosis in response to calcium ionophores. These findings suggest that citrullination mediated by PAD4 and NE-dependent protein hydrolysis of histones share common features but may play a key role in different situations.^144,156^

Activation of cell cycle and DNA repair signaling is also important in NETosis. The cell cycle protein-dependent kinase (CDK) 4/6 is activated during NETosis. CDK6 is required for NETosis, as a previous study has reported mice with CDK6-deficiency are more susceptible to infection. S-phase events (including DNA synthesis and histone gene transcription) are not found during NETosis, while M-phase events (laminin phosphorylation and centrosome segregation) are important the formation of NETs.^157^ These results suggest that neutrophils utilize the properties of the cell cycle to break down the nuclear membrane. Upon rupture of the nuclear membrane, the dispersed chromatin mixes with granule proteins in the cytoplasm to form NETs.

#### The non-lytic NETosis

The non-lytic NETosis, occurs through a NOX-independent pathway as known as ‘vital NETosis’, which can be induced by activated platelets, certain microbes, and calcium ionophore carrier A23187. It does not require ROS generation nor result in cell death and is especially critical for acute invasive infection. In contrast to lytic NETosis, neutrophils do not rupture and die, but rather excrete NETs to the outside of the cell by vesicular transport.^128^ In this pathway, neutrophils can release mtDNA to form NETs when stimulated by LPS or C5a. Furthermore, it has been illustrated that some pathogens can trigger a rapid non-lytic NETosis by activation of TLR2 and C3, such as *S. aureus* and *Candida albicans* (*C. albicans*).^109,123^ Moreover, platelets stimulated by LPS can also induce non-lytic NETosis by activating TLR4 in platelets. It is important to note that several studies have described a new formation of NETs containing mainly mitochondrial instead of nuclear DNA. Massive and fast release of mtDNA without loss of viability is detected in neutrophils primed with IL-5/IFNγ or LPS. Unlike the non-lytic NETosis containing nuclear DNA, mitochondrial NET formation depends on ROS, since ROS inhibitor treatment or utilization of neutrophils from patients with granulomatous diseases with ROS deficiency, could not release NETs. However, the detailed molecular mechanisms remain unclear.^109,156^

More importantly, these pathways of NET formation are not completely independent from each other. For example, acetylation modification of histones in NETs upregulates the immunoreactivity of NETs, and the use of low concentrations of deacetylation inhibitors promotes the formation of NETs, but when the dose is increased to a certain level, the NET formation is inhibited.^158^

Recently studies have shown that NETs formed by neutrophil subpopulations with varying densities play distinct roles in diverse pathologies. High-density neutrophils (HDNs) are typically found in healthy conditions, whereas low-density neutrophils (LDNs) are predominantly associated with pathological settings. LDNs can be co-segregated with the peripheral blood mononuclear cell fraction after centrifugation.^159^ LDNs often exhibit immunosuppressive effects and are prone to forming NETs. Elevated levels of LDNs have been observed in the blood of patients with systemic lupus erythematosus (SLE), antiphospholipid syndrome, and lung infections.^160–162^

### Molecular mechanisms regulating NET formation

#### Kinases in NET formation

**Fig. 3:**
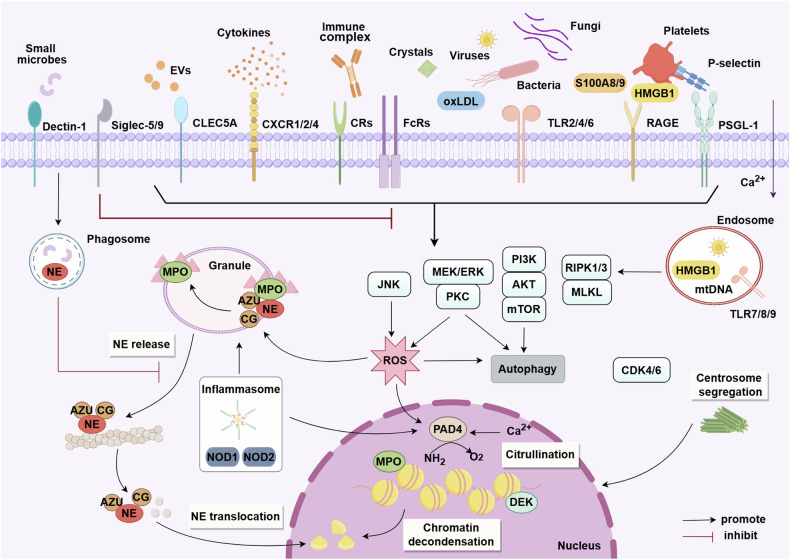
Molecular mechanisms regulating NET formation. The formation of NETs, also known as NETosis, can be initiated by microbial and endogenous stimuli. Various receptors, including those activated by immune complexes, bacteria, fungi, viruses, oxLDL, S100 calcium-binding proteins, and crystals, trigger NETosis via downstream effector proteins. Activated platelets can also induce NETosis through interaction between HMGB1-RAGE and P-electin-PSGL1. Signaling pathways such as MEK/ERK/PKC or JNK induce ROS generation, which is central to triggering NETosis by releasing NE from the azurosome complex. NE degrades the actin cytoskeleton and translocates to the nucleus to drive chromatin decondensation by processing histones. Additionally, chromatin decondensation can be promoted by MPO and DEK binding, as well as the activation of PAD4, which always employs calcium ionophores and mediates histone citrullination. NETosis also relies on CDK4/6 and the segregation of centrosomes. Autophagy and PI3K/AKT/mTOR signaling are also implicated in NET formation. NOD1/NOD2-linked signaling pathways may promote NET formation through both MPO-NE and PAD4 pathways. EVs can act as endogenous danger signals to induce NET formation by activating multiple receptors, including CLECs. Phagocytic receptors like Dectin-1 inhibit NETosis in response to small microorganisms by sequestering NE to phagosomes, while Siglec-5 and Siglec-9 suppress NETosis by limiting neutrophil activation. This figure was created with the assistance of Figdraw (www.figdraw.com)

Since 2020, increasing evidence has concentrated on the molecules involved in the regulation of NET formation, particularly kinases and receptors.^156,163^ The kinases implicated in NETosis include kinases activated by calcium influx, or cell cycle regulators, and cytokines involvement in downstream activation (Fig. 3). The protein kinase C (PKC), which is dependent of phospholipid and activated by ester and calcium, in particular PKCα, PKCβ1, and PKCζ, mediates NET formation induced by different stimuli.^164^ Dowey et al. have demonstrated that PKC inhibitor, ruboxistaurin, reduces pro-inflammatory and tissue-damaging consequences, as well as NET formation. Downey et al. have completed phase III trials for other indications without safety concerns.^165^ It is also important to clarify that PKCβ/δ/Cζ are all implicated in the oxidative burst, spreading and activation of NET formation by calcium ionophore A23187, whereas in PMA-activated NET formation, only PKCβ is associated with these functions.^164^ The regulator of cell cycle G1/S transition CDK6, and the Raf-MEK-ERK pathway are also critical for PMA-induced NETosis.^157^ In addition, receptor-interacting protein kinase (RIPK), and the mixed lineage kinase domain-like (MLKL) are involved in NET formation induced by antineutrophil cytoplasmic antibody (ANCA) and monosodium urate (MSU) crystals.^166,167^ Neutrophils from patients with chronic granulomatous diseases are unable to be phosphorylated by PMA-induced MLKL, while RIPK3 genetic depletion in mice blocks NET formation activated by MSU crystals.^167^

Oliveira et al. have identified that in response to different NET stimuli, phosphatidylinositol 3-kinase (PI3K) isoforms and related signaling partners can be mobilized, including inflammatory cytokines, growth factors, and chemokines. PI3Kα and PI3Kγ isoforms contribute to NET formation across multiple stimuli, whereas the involvement of other isoforms depends on stimuli. Some PI3K isozymes are found to signal through the typical downstream effector of PI3K, AKT, while others cannot. Downstream of PI3K, all stimuli can regulate NET formation with mammalian target of rapamycin (mTOR) and phospholipase C γ 2 (PLCγ2). Conversely, the participation of other kinases depends on the different stimuli, both tumor necrosis factor alpha (TNFα) and GM-CSF rely on pyruvate dehydrogenase kinase 1 (PDK1) and AKT, and TNFα relies on s6 kinase (S6K).^168^ In addition, the requirement for PI3K has also suggested the role of autophagy in NET formation, as it also relies on this enzyme. Consistent with this, in a bone marrow-specific murine model of autophagy deficiency, Bhattacharya et al. identified the significance of autophagy in neutrophil degranulation regulation. Neutrophils deficient of autophagy could inhibit degranulation of neutrophils by suppressing ROS production mediated by NADPH oxidase, indicating the correlation of NADPH oxidase with the impacts of autophagy on neutrophil degranulation.^169–171^ Autophagy inhibition (e.g., PI3K inhibitors) can result in a reduction of NET release, while its activation (e.g., rapamycin) enhances the formation of NETs.^172^ In addition, ROS can rapidly increase the pH value of primary vesicles and then induce autophagy, which is necessary but insufficient to induce NET formation.

Recently, c-Jun NH2-terminal kinase, and nonreceptor tyrosine kinase janus kinase (JAK), especially JAK2, have been implicated in NET formation.^173–175^ Jak2^V617F^ has been identified as one of the most common driven factors of myeloproliferative neoplasms. Mice carrying Jak2^V617F^ are more prone to NET and thrombus formation, while ruxolitinib, a clinically available JAK2 inhibitor, can eliminate the formation of NETs in a murine model of deep vein stenosis.^175^

#### Receptors in NET formation

Neutrophils recognize PAMPs or DAMPs when they are recruited to infectious sites, thereby activating specific surface receptors (Fig. 3). These receptors activate different intracellular signaling mechanisms to regulate a variety of neutrophil functions, including NET formation.

TLRs play a crucial role in recognizing host cells and responding to microbes. Except TLR3, all other TLRs are expressed on the surface of neutrophils in human. TLR2 and TLR4 are necessary in the induction of NOX-dependent NETosis by the fungus *Fonsecaea pedrosoi* (*F. pedrosoi*). In bacteria, *Wolbachia endobacteria (W. endobacteria)* can be recognized and initiate NETosis by TLR2 or TLR6. Furthermore, HIV-1 is captured and killed by NETs through the mediation of TLR7 and TLR8.^123,176^ In addition to pathogens, substances such as DAMPs, oxidized low-density lipoprotein (OxLDL), and activated platelets have been reported to promote NET formation via TLRs.^131,141,177^ Inhibition of TLRs can reduce NET formation, for example, TLR9 antagonist administration significantly abrogates NET formation, as well as cell death mediated by endoplasmic reticulum (ER) stress and induced by NETs.^178,179^

The cytoplasmic receptors, NOD-like receptors (NLRs), is the second line of defense against pathogens. Alyami et al. found that *Fusobacterium nucleatum* *(F. nucleatum)* upregulates the expression of nucleotide-binding oligomerization domain 1 (NOD1) and NOD2 to trig NET formation in a time-dependent manner.^180^ Another study on diabetic wound healing identified the role of NLRP3/Caspase-1/Gasdermin D (GSDMD) pathway in NET formation and release.^181^ Uptake or formation of cholesterol crystals in lysosomes can also cause membrane disruption, as well as activation of NLRP3 inflammasomes. Activation of inflammasomes in neutrophils cleaves GSDMD, followed by the formation of membrane pores and release of IL-1β and IL-18, ultimately resulting in pyroptosis or NET formation in hyperlipidemic mouse models.^182^

Immune cells (including lymphoid and myeloid cells) express a variety of C-type lectin receptors (CLRs) on their surface, for instance, L-selectin, macrophage inducible C type lectin (Mincle), macrophage inhibitory cytokine 1 (MIC1), of which Dectin 1 and Dectin 2 are usually expressed on neutrophils. The CLRs are able to recognize polysaccharides of microbial membranes directly and activate the immune responses by promoting the secretion of inflammatory cytokines and the formation of NETs. Numerous studies have reported that viruses may interact with lectins in immune cells via terminal glycan on their surface.^183,184^ Among members of the human CLRs, dendritic cell/lymphocyte-specific intercellular adhesion molecule-3-grabbing non-integrin (DC/L-SIGN), LSECtin, as well as spleen tyrosine kinase (Syk)-coupled C-type lectin member 5A (CLEC5A) and CLEC2, have been shown to play roles in virus-associated NET formation and inflammation.^185^ Stimulation of P-selectin upregulates the expression level of P-selectin glycoprotein ligand-1 (PSGL-1) and increases the phosphorylation of Syk, thus modulating NET formation in neutrophils.^186^ Sung et al. have illustrated that extracellular vesicles (EVs) from activated platelets can induce NET formation via activation of CLEC5A/TLR2 heterocomplex, while inhibition of CLEC5A and TLR2 by a bi-specific antibody almost completely abolishes NET formation-induced by EVs.^187^ Interestingly, besides being involved in NET formation, CLRs can inhibit the release of NETs as well. For example, Dectin-1 acts as a size sensor for microbial phagocytosis by neutrophils to prevent NETosis via blocking NE translocation to the nucleus.^122,188^

Complement receptors (CRs) are also mainly expressed on lymphoid and myeloid cells, and play an important role in the regulation of innate and acquired immune responses. There are specific interactions between complement factors that eliminate circulating antigens and clear apoptotic cells. One of the first evidence showing the importance of a complement system in NET formation is that neutrophils from mice with C3 deficiency have difficulty in NET formation,^189^ and those from mice with C3aR deficiency cannot form NETs either.^190,191^ To date, the most common CRs promoting NET formation are CR1, CR3, CR4 and CR5. In addition to CR1 antagonist, blocking of CR3 can inhibit NET formation in response to certain pathogens.^192,193^ A recent study has indicated that in neutrophils infected with SARS-CoV-2, the process of NETosis might be amplified by C5a/C5aR1 signaling, while treatment of neutrophils with DF2593A, a selective C5aR1 allosteric antagonist, inhibits NET formation, which provides a promising therapeutic strategy for COVID-19.^194^

RAGE is a multiligand transmembrane pattern recognition receptor, and its ligands include HMGB1, advanced glycation end products (AGEs), and the S100 family, etc. When activated, RAGE activates multiple intracellular signaling pathways and promotes the production of various inflammatory substances. HMGB1, by binding to RAGE, induces neutrophil activation and promotes the formation of NETs, a process that is dependent on the involvement of NADPH oxidase. The disulfide HMGB1 has also been observed in venous thrombosis to promote pro-thrombotic NET formation mediated by RAGE. More importantly, the employment of HMGB1-neutralizing antibodies eliminates NET formation.^195^ In the lupus-prone mice, NET formation in the glomerulus is remarkably suppressed in RAGE-deficient mice, along with the improvement of renal pathological scores, suggesting that the blockade of RAGE might be a promising therapeutic target for SLE.^196^ HBV-induced S100A9 accelerates the formation of NETs mediated by TLR4/RAGE-ROS signaling in hepatocellular carcinoma (HCC).^126^ In addition, S100 family calprotectins are also released upon the formation of NETs, shown as the failure of neutrophils from patients deficient in PMA-induced NETosis to release S100A8 or S100A9 in response to PMA stimulation, indicating that these calprotectins might amplify the activation of NET formation.^197^

Moreover, other receptors have also been shown to mediate NET formation. Multiple immune cells express Fc receptors (FcRs), thus driving humoral and cellular immune responses by facilitating the uptake of immune complexes. In one report, FcγRIIa directly participates in activation of NETosis, while another report demonstrates that FcγRIIa merely promotes phagocytosis and NET formation can be induced by FcγRIIIb through MEK/ERK signaling pathway.^198,199^ It remains unclear which receptor plays a major role or whether their interactions are critical for the formation of NETs. FcRs also seem to be involved in NET formation during infection of bacteria, as neutrophil exposure to ammoniated *S. aureus* suggests that activation of FcRs promotes NET release.^200^ In addition, neutrophil effector functions (e.g., degranulation and NETosis) are also reported to be mediated by chemokine receptors. Only CXCR1/2/4 have been identified to be implicated in NET formation to date.^200^ For example, CXCR1 and CXCR2 have been confirmed to be involved in mediating chemokines-promoted NETosis in tumors.^201^ CXCR2 induces NET formation by cooperating with PSGL-1, which signals the recruitment of neutrophils, thereby further promoting deep vein thrombosis.^202^ Overlapping subsets of immune cells express sialic acid-binding immunoglobulin-like lectins (Siglecs). Each Siglec binds to specific endogenous glycosylated glycan to initiate signaling programs and participate in cellular responses. Several Siglecs have been reported to play a regulatory role in NET formation, especially Siglec-9. Siglec-9 is considered as a neutrophil checkpoint and can suppress NETosis in inflammation and cancer immune evasion. Delivery of an artificial glycopeptide targeting Siglec-9 to the surface of intact cells could suppress NET formation and induce neutrophil apoptosis. A pair of receptors, Siglec-5 and Siglec-14, are expressed on monocytes and neutrophils, as Siglec-5 promotes bacterial survival through impairing NET formation, while Siglec-14 has opposing effects in the regulation of host immunity.^203,204^

## NETs in health

**Fig. 4:**
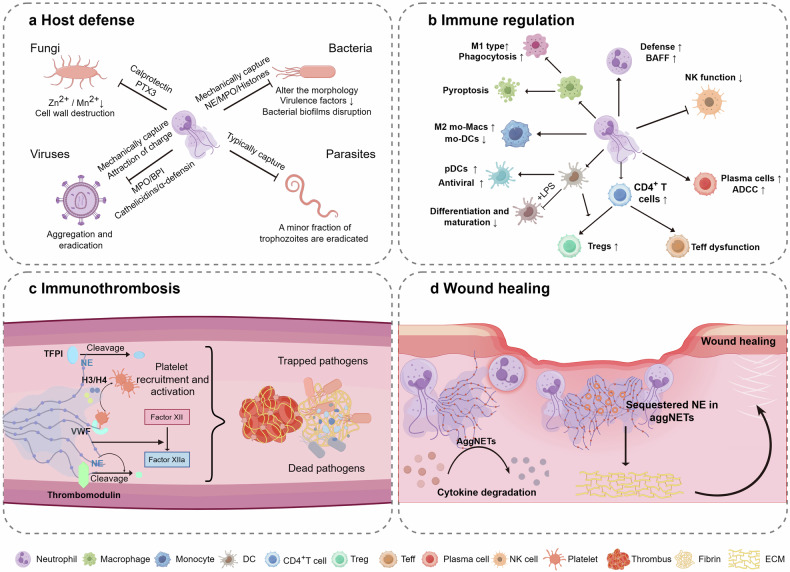
NETs in health. NETs play a crucial role in maintaining homeostasis. **a** NET function by capturing and immobilizing pathogens, relying on specific proteins embedded within the NETs to modify the morphological structure of these pathogens, thereby neutralizing and ultimately killing them. **b** NETs enhance neutrophil defense, promote macrophage polarization, induce pyroptosis, and facilitate pDC differentiation, thereby aiding antiviral functions. They also support CD4^+^ T cell and B cell activation while potentially impairing NK cell activity. **c** NETs promote immunothrombosis by activating factor XII, binding VWF, and triggering platelet activation via histones H3 and H4. They also inactivate anticoagulants and facilitate activation of the extrinsic pathway, aiding in pathogen defense. **d** AggNETs promote inflammation resolution and wound healing by degrading pro-inflammatory cytokines and sequestering NE to protect the extracellular matrix from proteolysis. This figure was created with the assistance of Figdraw (www.figdraw.com)

The bulk of materials associated with NETs are derived from the nucleus, resulting in a significant enrichment of core histones.^205^ Additionally, these materials contain elevated levels of cytosolic proteins such as S100 proteins, MPO, and granule proteins (NE and proteinase).^144^ The proteins contained within the reticular structure of NETs serve as the foundation for the physiological functions of NETs.^144,206^ NETs are integral components in the preservation of homeostasis, as evidenced by their involvement in host defense, immune regulation, immune thrombosis and wound healing, thereby serving beneficial functions to a certain degree (Fig. 4).^207–209^ Comprehending these physiological functions will aid in the formulation of more holistic clinical treatment strategies.^210^

### Host defense

As a foundational element of innate immunity, the primary function of NETs is to defend the host from pathogenic invasion (Fig. 4a).^20^ NETs effectively combat infections by ensnaring, immobilizing, and neutralizing a diverse array of pathogens, encompassing fungi, Gram-positive and Gram-negative bacteria, parasites and viruses.^144,211^ Neutrophils possess a distinctive microbe-detection mechanism, which enables them to customize their antimicrobial reactions towards pathogens based on microbial size.^212,213^ The ineffectiveness of phagocytosis in eliminating the large filamentous form of fungi highlights the necessity of NETs in effectively controlling these pathogens, particularly in individuals with MPO deficiency, leading to recurrent fungal infections.^214–216^

*Candida albicans*, a significant pathogen in invasive candidiasis, has been demonstrated to be effectively eliminated by calprotectin (S100A8/A9) within NETs in vitro and in vivo.^217,218^ This antimicrobial protein complex functions as a divalent metal ion chelator, exhibiting strong efficacy against a range of fungal pathogens such as *Candida albicans*, *C. neoformans*, and *Aspergillus spp*.^219^ Upon interaction, calprotectin demonstrates antifungal properties by sequestering Zn^2+^ and/or Mn^2+^, crucial elements for the growth of these pathogens.^197,220^ Moreover, NETs have the capability to alter the cell wall composition of *Candida albicans*, resulting in the exposure of β-glucan and increased detection by Dectin-1-positive immune cells.^221^ *Aspergillus spp* are widely distributed environmental fungi that emit spores, which are consistently inhaled but effectively eliminated by individuals with intact immune systems.^222^ As previously stated, calprotectin serves as a crucial antifungal agent in combating *Aspergillus spp* and has the ability to induce irreversible zinc deprivation at elevated concentrations.^214,223^ In a clinical investigation of chronic granulomatous disease patients undergoing gene therapy, the restored release of calprotectin is essential for protecting against *Aspergillus spp* and managing invasive pulmonary aspergillosis.^224^ NETs have also been observed to influence host immunity to *Aspergillus fumigatus* by releasing long PTX3, a pattern recognition receptor that triggers complement activation and aids in pathogen detection.^225^

The antibacterial properties of NETs continue to be a subject of scholarly discussion, with the potential for NETs to exhibit varying degrees of efficacy in the eradication of diverse bacterial strains.^20^ The morphological effects of NETs in bacterial infections represent a prominent and direct approach. NETs can alter the morphology of bacteria by ensnaring them with the web-like structure.^1,211,226^ Imaging techniques utilizing flow chamber systems or intravital microscopy effectively demonstrated the capture of *E. coli* by accumulated NETs in hepatic sinusoids during sepsis.^227^ In the absence of bactericidal elements, NETs capture pathogens without completely eliminating them, as they may not disrupt the structural integrity of bacterial cell walls or induce further alterations in bacterial morphology.^228–231^ Histones, which are rich in positively charged lysine and arginine residues, have been shown to exhibit bactericidal activity at low concentrations.^232,233^ Likewise, NE eradicates bacteria through the degradation of proteins located on the outer membrane of bacteria, while also focusing on the virulence factors specific to colonic enterobacteria.^234^ MPO continues to be active on the extruded NETs, producing ROS-like hypochlorous acid to kill bacteria.^211,235^ Additionally, NETs play a role in disrupting bacterial biofilms, which can also contribute to alterations in bacterial morphology.^236,237^ Interestingly, the environment in which NETs are formed affects their ability to kill bacteria. NETs formed under dynamic conditions trap more bacteria but kill them less effectively compared to those formed under static conditions.^228^

The mechanisms by which NETs defend against viral pathogens exhibit a range of diversity.^176,238^ First of all, the web-like structure can trap and immobilize viral particles, preventing their spread through electrostatic attraction.^239^ In addition to mechanically trapping, NETs also possess the ability to attract viral envelopes with negative charges, such as those found in influenza A particles, HIV-1, and norovirus, through the presence of positively charged amino acids. This process leads to the aggregation of these viruses, ultimately aiding in the containment and eradication of the pathogens.^239,240^ Furthermore, antimicrobial proteins such as MPO, cathelicidins, and α-defensin are attached to the chromatin backbone of NETs.^241,242^ These proteins have demonstrated antiviral activity against both enveloped and non-enveloped viruses.^124,239,243^ Additionally, the activity of human respiratory syncytial virus is also impeded by NETs, a phenomenon that may be associated with the presence of serine proteases and bactericidal permeability-increasing protein within NETs.^244,245^

A series of studies have shown that parasite infections can result in significant neutrophil infiltration and the production of NETs, although most parasites are typically captured but not entirely eradicated.^246^ In vitro formation of NETs has been documented as a mechanism capable of ensnaring *E. histolytica*; however, NETs do not impede its proliferation, with additional studies indicating that only a minor fraction of trophozoites are eradicated.^247,248^ Similarly, *Strongyloides stercolaris* and *Brugia malayi* can induce neutrophils to release NETs, which may help trap larvae but does not lead to their death in vitro.^249,250^ NETs cannot kill *Trypanosoma cruzi*, the cause of Chagas disease, but they can restrict its invasion and replication.^251^ Overall, the defensive protective role of NETs in parasitic infections remains poorly understood, potentially due to limited availability of experimental models for investigation.^20,246,252^

In this chapter, we focus on the reported host defense mechanisms related with NETs. Further research and discussion are needed to understand how NETs eliminate microbes. While NETs play a crucial role in combating infections, their tendency to trigger a systemic inflammatory response, referred to as the “waterfall effect,” can negatively impact host survival, particularly in viral infections.^253–255^ In cases of HBV-related acute-on-chronic liver failure, elevated NET levels are associated with poor patient outcomes.^256^ Similarly, excessive NET release in patients with COVID-19 contributes to complications such as coagulopathy and lung damage.^127,257,258^ These pathological effects are discussed in detail in subsequent sections. Therefore, precise control over the production and breakdown of NETs is imperative in order to mitigate pathogenic inflammation.

### Immune regulation

Recent studies suggest that while NETs are part of the innate immune system, they also play a significant role in modulating the functions of various immune cells (Fig. 4b).^42,206,259^ In light of the crucial role of immune homeostasis, it is essential to comprehensively investigate the interplay between NETs and both adaptive and innate immune responses.^260^

Neutrophils exposed to isolated NETs activate various neutrophil functions in a concentration-dependent manner, according to several studies.^130,261,262^ These functions include the induction of granule exocytosis, generation of ROS and the NADPH oxidase NOX2, formation of NOX2-dependent NETs, increased phagocytosis, and eradication of microbial pathogens. Additionally, it has been observed that the activation of neutrophils by NETs involves pathways that entail the phosphorylation of p38 Akt/ERK1/2. Collectively, NETs stimulate neutrophil effector function and bolster antimicrobial defense. Moreover, NETs possess the capacity to connect the adaptive and innate immune responses through the stimulation of B-cell Activating Factor (BAFF) from neutrophils.^262–264^

The plasticity of macrophages renders them essential in the immune response to pathogens, tissue regeneration, and the preservation of homeostasis.^265^ Studies have demonstrated that the DNA component of NETs contributes to the activation and polarization of pro-inflammatory macrophages via the TLR9/NF-κB signaling pathway.^266,267^ In a separate study, it was observed that the levels of iNOS, CD80, and CD86, markers associated with M1 macrophages, were markedly elevated following treatment with NETs. Conversely, the expression of CD206, an M2 marker, was significantly reduced.^268^ Additionally, NETs aid in the transfer of antimicrobial peptides by macrophages, thereby augmenting their antimicrobial capabilities.^259^ It is important to acknowledge that NETs have the potential to induce caspase-1-dependent pyroptosis in macrophages via HMGB1.^269^ This interaction additionally aids in combating extracellular pathogens.^270^ Upon exposure to *Staphylococcus aureus*, *Streptococcus pneumoniae*, and *Pseudomonas aeruginosa*, it was observed that NET formation enhances antimicrobial efficacy by promoting macrophage phagocytosis and facilitating the transfer of neutrophil-specific antimicrobial peptides to macrophages.^270–272^ These findings underscore the importance of the crosstalk between NETs and macrophages in achieving optimal bactericidal activity through NET formation.

NETs have a dual impact on the function of dendritic cells (DCs).^273^ They attract DCs and stimulate them through the IgG Fc fragment via the IIa receptor with low affinity (FCγII), resulting in the generation of interferon-alpha (IFN-α) through TLR9.^274^ Specific granule proteins found in NETs, such as MPO, HMGB1, and secretory leukocyte proteinase inhibitor (SLPI), stimulate plasmacytoid DCs (pDCs) to produce antiviral factor.^275^ Furthermore, pDCs have the capacity to induce the differentiation of naïve CD4^+^ T cells into Th17 and Th1 cells subsets.^133,276^ However, it has been observed that NETs have the potential to impede the differentiation and maturation of DCs in response to LPS stimulation.^277^ Moreover, the treatment of immature DCs with NE resulted in the generation and secretion of transforming growth factor beta (TGF-β), which in turn facilitates the differentiation of regulatory T cells (Tregs).^278^

Monocytes possess the capability to undergo differentiation into either DCs (mo-DCs) or macrophages (mo-Macs), with the balance between the mo-DC and mo-Mac fate being subject to adjustable homeostasis.^279,280^ Furthermore, the incorporation of NETs into monocytes treated with interleukin-4/granulocyte-macrophage colony-stimulating factor (IL-4/GM-CSF) resulted in the downregulation of IL-4 receptor on monocytes, hindering their full differentiation into DCs while promoting their differentiation into M2 macrophages.^281^ mo-DCs are a significant contributor to the progression of pathogenic processes in chronic inflammation. Consequently, NETs serve a crucial function in regulating immune homeostasis.^282–284^

Natural killer (NK) cells, a significant subset of innate immune cells, are known to have their function predominantly suppressed by NETs.^260^ The addition of DNase I to degrade NETs in postoperative immunotherapy for HCC has been shown to enhance the infusion of NK cells and reduce the risk of HCC recurrence, indicating a potential alleviation of the inhibitory effects of NETs on NK cell activity.^285^ RNA-Seq analysis demonstrated that NETs impede NK cell function via the interaction with carcinoembryonic antigen-related cell adhesion molecule 1 (CEACAM1) during the host’s antiviral immune response.^286^ Furthermore, in a murine model where NET formation was disrupted, a decrease in dNKs was observed.^287^

The T cell receptor serves as a crucial mechanism for NETs to engage with T cells, leading to a reduction in T cell activation threshold and enhancement of antigen-specific immune responses.^288^ Research has shown that Toxoplasma gondii-induced NETs enhance the recruitment of CD4^+^ T cells and the secretion of TNF, IFN-γ, and IL-6, suggesting that the adaptive immune response is partially enhanced by NETs.^289^ Notably, CD4^+^ T cells exposed to NETs demonstrate elevated levels of activation markers, including CD69 and CD25. A comparable pattern of activation marker expression is noted in CD8^+^ T cells subsequent to exposure to NETs.^259,290^ Furthermore, NET-associated histones have the capacity to induce the differentiation and cytokine production of Th17 cells through a TLR2/MyD88/STAT3/RORγ-dependent pathway.^291^ It is imperative for bolstering immunity against fungal and bacterial infections, as well as enhancing anticancer immunity.^260^ While another study concluded that Tregs are modulated by NETs, which enhance mitochondrial oxidative phosphorylation and support the differentiation of Tregs from naïve CD4^+^ T cells through TLR4 signaling.^39^ NETs may also enhance antiviral adaptive immunity by lowering the activation threshold of T lymphocytes.^242^ In summary, NETs have been observed to promote T cell activation, proliferation, and differentiation, thereby modulating adaptive immune responses during periods of necessity.

B cells, another important responder to adaptive immunity, have been identified as associated with NETs, in addition to macrophages, DCs, NK cells, and T cells.^229,259,260^ Upon encountering antigens, B cells undergo rapid proliferation, with the majority of cells differentiating into plasma cells (effector B cells) and generating antibodies. LL37-DNA complexes originating from NETs have been found to possess the distinctive capability of localizing to endosomal compartments within B cells and inducing polyclonal B cell activation through TLR9, as well as selectively amplifying self-reactive memory B cells that generate anti-LL37 antibodies in response to antigens.^292,293^ In addition, citrullinated histones are recognized as a classic antigen for B cell activation, and the MAPK-p38 pathway represents an additional mechanism through which NETs induce B cell activation.^294,295^ B cells play a crucial role in mediating humoral immune responses, as their activation is necessary for antigen presentation, antibody-dependent cell-mediated cytotoxicity against tumors, as well as antibacterial and antiviral activities.^296–299^ Hence, it is possible that the beneficial effects of these functions on health conditions could be further augmented following exposure to NETs.

NETs are essential in maintaining immune homeostasis, but they also activate immune cells such as B cells, antigen-presenting cells, and T cells, contributing to autoimmune diseases including RA, ANCA associated vasculitis (AAV), SLE, and antiphospholipid syndrome.^109,300^ In tumors, NETs create an immunosuppressive environment that weakens the antitumor immune response of macrophages, CD4^+^ T, and CD8^+^ T cells, thereby accelerating cancer progression and metastasis.^39,301,302^ Notably, the impact of NETs on immune cells varies between tumor and non-tumor settings.^260^ Additional specific details will be provided in subsequent sections.

### Immunothrombosis

Researchers introduced the term immunothrombosis, prompting a shift in contemporary research towards investigating its potential protective role in the context of infection.^13^ To uphold homeostasis and bolster the host defense against infectious pathogens, the innate immune system initiates local coagulation, leading to microvascular thrombosis, a process that is dependent on neutrophils and NETs (Fig. 4c).^9^ The development of thrombi is initiated by the interaction of activated neutrophils and monocytes infected with pathogens, as well as activated platelets and coagulation factors. This process serves a protective role by restricting, sequestering, and eliminating pathogens, and can manifest in veins, arteries, and microvessels across various anatomical levels.^303,304^

NETs contribute a cell specific mechanisms to potentiate immunothrombosis.^9–12^ NETs can bind to and activate platelets, forming a platform that boosts neutrophil elastase activity and promotes coagulation.^304^ NE on NETs degrades and inactivates Tissue factor pathway inhibitor (TFPI), with help from activated platelets that aid in NET formation. Neutrophil serine proteases facilitate the activation of coagulation by tissue factor, known as the extrinsic pathway. This process allows platelet-neutrophil conjugates to directly stimulate coagulation by increasing intravascular tissue factor activity. Thrombomodulin may undergo degradation via cleavage by NE and inactivation by neutrophil oxidases in NETs. Factor XIIa can be formed during fibrin formation when extracellular nucleosomes within NETs activate the contact pathway of coagulation. Additionally, histone components in NETs can induce thrombosis by activating platelets through TLR2 and TLR4.^13^ Platelets directly interact with neutrophils in response to bacterial products, inducing the formation of NETs through a process known as NETosis.^12^ Additionally, the histone components of NETs, specifically histones H3 and H4, have been found to influence platelets by promoting their recruitment and activation.^305,306^

Immunothrombosis has been proposed to fulfill a minimum of four distinct physiological roles.^13,303,306^ Firstly, it aids in the capture and entrapment of circulating pathogens, thereby restricting their spread by confining them within the fibrin network. As a second benefit, microthrombi resulting from immunothrombosis in microvessels inhibit tissue invasion by pathogens. Thirdly, the blood clots create a distinct space that enhances the concentration of antimicrobial strategies and their targets, thereby promoting pathogen eradication. Four, microvascular buildup of fibrinogen or fibrin attracts more immune cells to the infected or damaged tissue, enhancing pathogen recognition and immune response coordination.^13^ In conclusion, immunothrombosis with NETs helps identify, contain, and eliminate pathogens to protect the host without causing harm.^303^ Therefore, it has been argued that universal use of anticoagulation in these patients cannot be recommended.^307^

It is imperative to acknowledge that uncontrolled immunothrombosis can lead to disseminated intravascular coagulation (DIC), especially during sepsis, and increases the risk of thrombosis and cardiovascular issues in individuals with chronic inflammatory or infectious conditions.^9,308^ The protective phase of immunothrombosis should be rigorously evaluated from a clinical perspective.

### Wound healing

Many studies view the role of NETs in wound healing negatively, but there is this is a controversial finding.^209^ It has been documented that aggregated NETs, which contain a diverse array of enzymes, have the potential to act as inflammatory mediators by degrading pro-inflammatory cytokines and chemokines, thereby promoting inflammation resolution and wound healing.^309–311^ Furthermore, aggregated NETs (aggNETs) have the ability to sequester NE and shield the extracellular matrix (ECM) from NE-mediated proteolysis.^309^ Bicarbonate-induced aggregated NETs have been observed to encapsulate necrotic regions and wounds. It is evident that aggregated NETs fulfill distinct functions in the context of wound healing compared to other forms of NETs (Fig. 4d).^312^ Previous research, particularly in diabetic patients, has primarily focused on the association between impaired wound healing and elevated levels of NETs-related proteins. Excessive or persistent NETs have been observed to contribute to delayed healing of diabetic foot ulcers, a topic that will be further detailed subsequently.^313,314^ In other words, research on the intrinsic mechanisms of different types of NET formation in wound healing is still in its early stage due to the diverse nature of wound formation and healing processes, as well as the various pathways that trigger NET formation.^209^

In conclusion, NETs are crucial for an antimicrobial defense mechanism within the innate immune system, functioning both as a physical barrier to impede the dissemination of pathogens and inflammatory mediators, and as a means to eliminate microbes through the action of extracellular DNA, citrullinated histones, and enzymes.^211,214,226,238^ Furthermore, the inflammatory nature of NETs serves to modulate the immune response and activate additional immune cells.^205,260,290^ NETs exhibit a tendency to aggregate at high neutrophil densities, degrade soluble inflammatory mediators through NET-associated serine proteases, thereby facilitating the resolution of inflammation and tissue regeneration.^209,313^ It is noteworthy that NETs serve a crucial function in preserving host well-being and physiological equilibrium.

## NETs in various diseases

### Infectious diseases

**Fig. 5:**
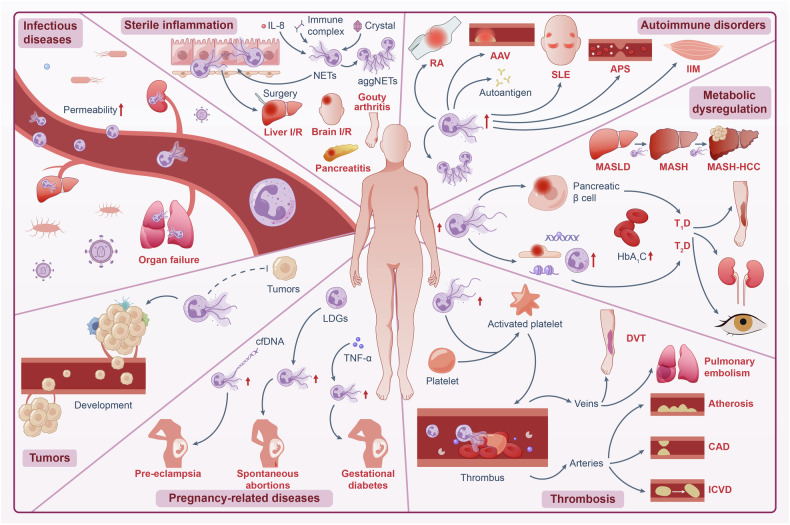
NETs in diseases. NETs are involved in various human diseases. NETs are central to the immune response against infectious agents, yet their role can be linked to a double-edged sword due to their potential to exacerbate tissue damage under conditions of sustained inflammation or persistent stimuli. NETs are implicated in a spectrum of nonpathogenic diseases, including sterile inflammation, autoimmune disorders, metabolic dysregulation, thrombosis, pregnancy-related diseases, and tumors, when dysregulated. Under sterile conditions, various stimuli, such as IL-8, immune complexes, and crystals, can facilitate the formation of NETs, leading to conditions like gouty arthritis. AggNETs facilitate the resolution of sterile inflammation. NETs are also implicated in pancreatitis and I/R injuries such as brain and liver I/R. In autoimmune disorders, beyond their pro-inflammatory function, NETs have emerged as potential autoantigens, contributing to the production of autoantibodies. NETs contribute to the disease process of T1D, while further investigation is required for their involvement in T2D. Circulating NET markers positively correlate with glycated HbA1c levels and the severity of diabetic complications. Additionally, NETs promote the progression of MASLD, from steatosis to MASH-HCC. NETs are also implicated in both venous (DVT and pulmonary embolism) and arterial thrombotic events (atherosclerosis, coronary artery disease, and ischemic stroke). Furthermore, NETs are associated with several pregnancy-related diseases, such as pre-eclampsia, spontaneous abortions, and gestational diabetes, contributing to their pathogenesis. The protumorigenic role of NETs in various cancers has been confirmed, although a bidirectional interplay between cancer cells and NETs is proposed. This figure was created by Adobe Illustrator Artwork 16.0 (Adobe Systems, USA)

As elucidated previously, NETs unequivocally play an essential role in orchestrating the immune response against infectious agents, notably by helping neutrophils immobilize, capture, and kill invading pathogens such as Gram-negative and Gram-positive bacteria,^3,4^ virus,^126,172,257^ fungi,^214,217,315^ and parasites.^316,317^ Impaired NET function may promote pathogens’ escape from the immune system and provide a niche for chronic infection.^16–18^ Nevertheless, akin to a double-edged sword, the sustained presence of inflammation or persistent stimuli can precipitate excessive NET formation, thereby exacerbating tissue damage in instance of inappropriate inflammation (Fig. 5).

While NETs effectively ensnare pathogens, certain pathogens have developed mechanisms to evade this process. Various pathogens, encompassing a spectrum including *V. cholerae*, *Streptococcus*, *Staphylococcus genera*, *P. aeruginosa*, *N. gonorrhoeae*, *M. tuberculosis*, *N. brasiliensis*, *Plasmodium*, *Mycoplasma*, *Leishmania*, and *Leptospira*, produce both endogenous and secreted endonucleases. These enzymes degrade the extracellular DNA scaffold of NETs, thereby dismantling and circumventing the entrapment.^207,318–320^ This evasion facilitated by endonuclease promotes subsequent invasion and dissemination from primary sites to distant organs and the circulation,^319^ which contributes to the exacerbation of inflammatory pathological conditions, including sepsis.

Sepsis represents a condition characterized by lethal dysfunction of multiple organs and is associated with a high rate of morbidity and mortality.^130,321^ During the early stages of sepsis, neutrophils are recruited from the blood to the infection site and release NETs.^208,322^ Studies have elucidated that dysregulated NET function during the early stages of infection contributes to the persistent systemic inflammation that initiates the development of sepsis.^16,130^ In contrast, as sepsis progresses, excess NETs damage tissue, increase vascular permeability and promote organ failure.^16,93,322,323^ Circulatory NETs in the bloodstream were significantly elevated and NET markers were also increased in patients with sepsis.^324–327^ A growing body of evidence reveals that in sepsis and acute injury, NET-bound histones are cytotoxic because of their ability to compromise cell membrane integrity.^328,329^ Meanwhile, other NET proteins, such as defensins and NE can disrupt cell junctions.^20,317^ In murine models of sepsis, a study observed marked platelet aggregation, thrombin activation, and fibrin clot formation within NETs in vivo.^330^ Aggumated accumulated NETs contribute to the sustained hyper-immunothrombosis in sepsis, which leads to lethal DIC complications in patients.^131,303,331^

NETs are regarded as the main players in antiviral immunity.^15^ Neutrophils and NETs have been reported to have protective effects in the early stage of viral hepatitis.^332,333^ A study indicated that NET release was decreased in patients with chronic HBV infection, and correlated negatively with hepatitis B surface Ag, hepatitis B E Ag, and hepatitis B core Ab levels.^333^ Nevertheless, HBV C protein and HBV E protein might inhibit the release of NETs by decreasing ROS production and autophagy.^333^ This suggests that impaired NET function may promote viral escape from the immune system and provide a niche for chronic hepatic virus infection. However, in HBV-related acute chronic liver failure (ALF), circulating neutrophils display a significantly heightened propensity to form NETs, which is closely associated with adverse patient outcomes.^256,334^ Excessive generation of NETs is widely acknowledged as a mediator of further pathophysiological abnormalities following SARS-CoV-2 infection.^335–337^ Elevated NET release has been documented in numerous patients with COVID-19, contributing to detrimental coagulopathy, immunothrombosis, and pulmonary endothelium damage within the alveoli.^257,335,338^ Inhibition of NETs in patients with COVID-19 has been shown to mitigate thrombotic tissue damage associated with COVID-19-related acute respiratory distress syndrome (ARDS) and mortality.^338–340^ Moreover, NET-derived histones have been identified in bronchoalveolar lavage fluid from patients with ARDS,^340^ underscoring the pivotal pathogenical role of NETs in lung injury.

In the context of infectious diseases, NETs exhibit dual roles. During the initial phases of infection, their normal function aids in pathogen clearance and prevents the transition of inflammation into a chronic state. However, in conditions such as sepsis and acute injury, NETs assume a detrimental role, compromising cell membrane integrity, exerting cytotoxic effects on epithelial and endothelial cells, and contributing to immunothrombosis formation.^303,329,341^ NET-mediated damage may exacerbate rather than constrain certain infections during chronic inflammation. Consequently, strategies aimed at optimal NET inhibition at pertinent disease stage represent potential strategies for infection management.

### Sterile inflammation

In contrast to pathogen-targeted mechanisms, sterile-associated NETs may entail heightened deleterious effects.^20,128^ Under sterile conditions, NET formation can be facilitated by various stimuli including but not limited to IL-8,^22^ immune complexes,^23^ crystals,^24^ or DAMPs, such as HMGB1.^25^ The deleterious impact of NETs on tissues manifests through direct cytotoxicity towards epithelial and endothelial cells, thereby potentiating tissue inflammatory cascades.^342,343^ Additionally, the influence of NETs extends to the modulation of inflammatory cytokines either through direct or indirect impact on diverse immune cell populations.

In sterile crystal-mediated inflammation, microcrystals including monosodium urate (MSU), calcium pyrophosphate dihydrate, calcium carbonate, calcium phosphate, calcium oxalate, and cholesterol can stimulate neutrophils to release NETs.^310,344^ Crystals of MSU monihydrate in joints and soft tissues elicit an acute inflammatory condition commonly known as gouty arthritis.^345^ Within the joint, MSU crystals instigate the release of inflammatory mediators, orchestrating the recruitment of neutrophils and subsequent NET formation.^167,346,347^ Infiltrated NETs contribute to the acute, profoundly painful, and tissue-damaging inflammation observed within the joints.^344^ NET formation in MSU crystal-induced arthritis is influenced by diverse factors, including the presence of inflammatory cytokines such as IL-1β.^348^ Neutrophils demonstrate increased release of NETs in response to synovial fluid from patients with gout, albeit partially abrogated by the IL-1β antagonist.^349^ Conversely, studies have unveiled that the excessive accumulation of aggNETs facilitates the resolution of gouty inflammation by encapsulating MSU crystals, degrading cytokines and chemokines, and inhibiting neutrophil recruitment and activation.^310,350,351^ These findings highlight the potential role of aggNETs as a mechanism promoting the spontaneous resolution of gout, thereby presenting novel therapeutic avenues. However, the precise underlying mechanisms are not fully understood.

Within the milieu of atherosclerosis (AS), circulating cholesterol form monohydrate cholesterol crystals, thereby fostering the formation of atherosclerotic lesions.^352,353^ These cholesterol crystals serve as potent inducers of NET formation, and in concert with cholesterol crystals, NET augment the release of cytokines released from macrophages via the IL-1/IL-17 and NF-κB signaling pathways.^24^ NETs have been discerned within the luminal regions of murine and human atherosclerotic lesions, as well as arterial thrombi, implying the potential NET formation across all stages of AS progression.^354–358^ Notably, within an atherosclerosis mouse model deficient in NE and proteinase 3 (PR3), NETs fail to generate, consequently exhibiting diminished plaque size.^24,359^ Collectively, NETs-derived extracellular components exhibit cytotoxic and pro-inflammatory attributes, culminating in cellular malfunction and tissue injury, thereby suggesting a nexus between lipid metabolism, inflammatory immunity, and atherosclerosis.^360^ In patients with suspected or established coronary artery disease, heightened levels of dsDNA and MPO-DNA complexes in plasma demonstrate a positive correlation with both the severity and quantity of atherosclerotic vessels.^361,362^ Consequently, strategies aimed at inhibiting NET release or the dissolution of NETs may present a promising therapeutic avenue in the context of NET-mediated AS and thrombosis.

In pancreatitis, studies substantiated that bicarbonate ions alongside calcium carbonate crystals can elicit the formation of aggNETs within the ductal tree via a PAD4-dependent signaling pathway.^344,363^ Besides their implication in the inflammatory insult to the pancreas, the presence of aggNETs within pancreatic ducts can precipitate catheter obstruction and foster the onset and progression of severe acute pancreatitis (SAP).^363^ Histological analyses of tissue specimens and pancreatic juice samples obtained from patients with pancreatitis have revealed the presence of aggNETs.^363^ A study suggests a fundamental role of NETs in gallstone formation, with inhibition of NET formation demonstrating efficacy in inhibiting gallstone development in vivo.^364^ Administration of DNase I to mouse models resulted in a marked reduction in neutrophil infiltration and tissue damage within the pancreas.^365^ Cumulatively, NETs exacerbate biliopancreatic duct obstruction and exacerbate inflammation, culminating in the manifestation of SAP. Furthermore, NETs contribute to multi-organ injury, infected pancreatic necrosis, sepsis, and thrombotic events associated with SAP.^365,366^

The involvement of NETs in ischemia/reperfusion (I/R) injury has generated recent attention. The reperfusion subsequent to abrupt blood flow restoration frequently triggers cerebral IR injury following an episode of cerebral ischemia.^367^ Neutrophils are prompted to release NETs in response to various stimuli, including platelet activation and the presence of IL-8, DAMPs, and TNF-α subsequent to ischemic stroke.^368^ The accumulation of NETs exacerbates inflammatory processes, thrombus formation, and neuron apoptosis.^369,370^ Constituents of NETs, such as MPO, histones, and other enzymes contribute to the leakage of blood-brain barrier. Furthermore, in individuals afflicted with ischemia-induced Alzheimer’s disease, heightened levels of amyloid-β (Aβ) precipitate platelet activation, leading to release of HMGB1 and subsequent NET formation, exacerbating disease progression.^371,372^ Notably, inhibition of NETs has been confirmed to facilitate neovascularization,^373,374^ indicating a potential therapeutic avenue in mitigating ischemic injury. The pro-inflammatory function of NETs has also been substantiated in liver I/R injury, exacerbating the inflammatory response and liver injury subsequent to I/R.^100,375,376^ DAMPs emanating from stressed hepatocytes, such as HMGB1 and IL-33 released from liver sinusoidal endothelial cells, serve as pivotal instigators for neutrophil infiltration and subsequent NET formation.^375,377,378^ Moreover, membrane-nonpermeable superoxide generated during I/R implicated TLR-4 signaling pathway activation, which subsequently instigated NOX and subsequent NET formation.^379^ Remarkably, interventions such as DNase treatment or inhibition of PAD4 have demonstrated considerable efficacy in mitigating liver inflammation in liver I/R.^377^

The similarity of NETs in infectious diseases and sterile inflammation lies in their dual role of both protecting and causing harm. In infectious diseases, NETs help clear pathogens and prevent chronic inflammation but can also cause cytotoxicity and contribute to immunothrombosis in conditions like sepsis. Similarly, in sterile inflammation, NETs, triggered by stimuli such as IL-8 and DAMPs, can cause direct cytotoxic effects on epithelial and endothelial cells, exacerbating tissue inflammation. In both scenarios, NETs can have beneficial and harmful effects on tissues and overall health.

### Autoimmune disorders

Accumulating evidence from in vitro, in vivo and clinical diagnostics suggests significant involvement of NETs in the pathogenesis of various autoimmune disorders, including but not limited to RA, AAV, SLE, and antiphospholipid syndrome (Fig. 5). NETs have emerged as potential disruptors of self-tolerance, serving as reservoirs of autoantigens that contribute to the production of autoantibodies characteristic of autoimmune disorders.^380,381^ Additionally, components of NETs are implicated in exacerbating the inflammatory milieu by facilitating complement activation and activaion of other specific immune cells, such as B cells and antigen-presenting cells, thus perpetuating the autoimmune responses.^292,382–385^

RA represents as a chronic systemic disease characterized by progressive joint inflammation and variable extra-articular manifestations.^386^ Central to its pathology are the anti-citrullinated protein antibodies (ACPAs), which exhibit high specificity for RA and can instigate the formation of pathogenic immune complexes within the affected joints.^387,388^ Neutrophils are abundant in the inflamed joints of patients with RA, displaying an augmented propensity for spontaneous NET formation.^389–392^ Moreover, this propensity for NET generation escalates upon stimulation with RA synovial fluid and ACPA-positive RA serum.^389,392^ Elevated levels of MPO-DNA complexes and cell-free nucleosome are observed in the serum of patients with RA,^393,394^ with their concentrations correlating with clinical parameters and ACPA titers in patient sera.^389,393,395,396^ Accumulated NETs release novel autoantigens, including citrullinated histones, which may further fuel the autoimmune response in RA.^389,397^ ACPAs have been reported to recognize autoantigens presented on NETs, especially the citrullinated histones.^398–400^ Additionally, NETs have been implicated in disrupting the cartilage structure and facilitating its citrullination, thereby exacerbating synovial inflammation.^401^ Overall, NETs play a central inflammatory role in RA and represent a significant source of autoantigens capable of eliciting pro-inflammatory responses within various organs, including the lungs and synovium, in patients with RA.^129,402,403^ Furthermore, NETs and NET-derived products hold promise as biomarkers for RA disease activity.

AAV represents a group of disorders characterized by inflammation and destruction of small and medium vessels, with autoantibodies against MPO and PRTN3 as key distinguishing markers.^404,405^ PRTN3 is expressed on the membrane of resting neutrophils, whereas MPO is stored within the granules, both of which are notably enriched within the NET structure.^300,406,407^ Analogous to RA, neutrophils in patients with AAV exhibit a heightened capacity for NET synthesis.^408,409^ In turn, NETs may be a key origin of ANCA-autoantigens.^408,410^ Some studies confirm that release of NETs may be triggered by a response to ANCA stimulation.^411,412^ Beyond their antigenic role, NETs exert influence on AAV progression by directly inflicting vessel damage through the cytotoxic release of NET-associated histone.^413^ Importantly, NET structures have been identified within various tissues from patients with AAV, promoting inflammation in multi-organs.^414,415^ Elevated levels of MPO have been detected in patients with AAV compared to those in remission.^416,417^ In mouse model with AAV, inhibiting PAD4-mediated NET formation has shown promise in reducing disease severity, indicating a potential therapeutic avenue.^417^ Thus, NETs may serve as novel biomarkers for disease diagnosis and represent promising targets for future therapeutics of AAV.

SLE is a systemic autoimmune disease characterized by pervasive inflammation across many organs.^418^ NETs represent a central origin of SLE autoantigens.^419,420^ Neutrophils sourced from healthy individuals exhibit a heightened propensity for NET formation when exposed to serum or plasma derived from patients with SLE, SLE–SLE-associated immune complexes and autoantibodies reciprocally fostering NET generation.^23,421^ The compromised clearance of NETs contributes substantively to SLE pathogenesis by extending the exposure duration of autoantigens and elevating levels of SLE-associated autoantibodies.^420,422,423^ Non-degraded NETs precipitate activation of the complement system, thus perpetuating inflammatory cascades.^424^ Within the SLE milieu, LDNs demonstrate augmented presence in circulation, with their levels correlating with distinct disease manifestations such as vasculopathy, skin disease, nephritis, and cardiopathy.^160,382,425,426^ Notably, these specific neutrophils exhibit increased spontaneous NET formation.^427^ Neutrophils from patients with SLE, particularly LDNs, display enhanced ex vivo NET formation, characterized by elevated levels of modified autoantigens and immunostimulatory molecules within the NET structure compared to those from healthy individuals.^23,421^ LDNs have been implicated in directly compromising endothelial cell integrity through the NET product MMP-9.^428^

NETs have also been implicated in other autoimmune disorders including but not limited to antiphospholipid syndrome,^429–432^ idiopathic inflammatory myopathies,^433–435^ multiple sclerosis,^436,437^ psoriasis,^438,439^ and inflammatory bowel diseases.^440,441^ Diverse autoantibodies have been shown to directly induce NET formation, with resultant NETs reciprocally promoting the production of autoantibodies. On one hand, NETs exhibit the capacity to directly inflict tissue damage, while on the other hand, they serve to catalyze the initiation and perpetuation of systemic autoimmune disorders, orchestrating intricate inflammatory responses by direct or indirect interactions with other immune cells. Collectively, escalated NET formation coupled with decreased NET degradation contribute to heightened levels of these structures and augmented exposure to modified autoantigens, thereby exacerbating tissue damage in these autoimmune conditions. Clinical interventions ought to ideally focus on selectively modulating dysregulated NET activity while keeping other essential antimicrobial functions.

### Metabolic dysregulation

Metabolic diseases such as diabetes mellitus (DM) and its associated complications pose a significant threat to public health, leading to diminished health and quality of life.^442,443^ The prevalence of DM is steadily increasing in both developing and developed countries, reaching epidemic proportions.^444–446^ Type 1 diabetes (T1D) necessitates insulin and involves the destruction of a significant number of insulin-producing pancreatic β cells, stemming from a chronic and progressive autoimmune dysfunction.^446^ Type 2 diabetes (T2D) represents a metabolic syndrome marked by reduced insulin sensitivity and impaired insulin production.^447^ The expression of PAD4 is elevated in neutrophils of patients with both T1D and T2D,^448^ and these neutrophils exhibit increased susceptibility to NETosis when stimulated in vivo.^449^ NET formation has been observed in the murine model with T1D,^450^ and clinical data similarly showed that NETs are elevated in patients with T1D.^451–453^ A recent study demonstrated a significant increase in circulating NE and PR3 levels in patients with T1D, strongly correlated with β cell autoimmunity, indicating a potential role of NETs in the onset and pathogenesis of the disease.^451^ Increased formation of NETs is associated with gut permeability in individuals with T1D, but not T2D.^454^ Further, NETs caused by gut leakage can trigger autoimmune response in non-obese diabetic mice.^455^ Improving gut barrier function via intestinal NETs degradation can prevent T1D in node mice.^456^ Early inhibition of NE finally resulted in decreased incidence of T1D in murine model.^457^ NETs can stimulate cytokine production and promote the generation of IFNγ-producing T cells in samples from T1D patients.^276^ Inhibition NET formation prevents the onset of diabetes in non-obese diabetic mice.^458^ Furthermore, NET inhibition alleviates vascular dysfunction in T1D mice.^459^ Based on these results, we posit that akin to autoimmune conditions discussed above, NET might similarly assume an antigenic function in the etiology of T1D, notably triggering the autoimmune disorders in the pancreas. Moreover, NETs may further contribute to systemic inflammation and complications in the progression of T1D.

A diverse array of circulating NET markers, including cell-free DNA, nucleosome DNA, and neutrophil expression of PAD4, have been reported to exhibit elevation in the circulation of individuals with T2D.^449,460,461^ These circulating NET markers have been observed to positively correlate with the level of glycated hemoglobin A1c.^462^ Nevertheless, the impact of hyperglycemia on NET formation remains controversial. Neutrophils isolated from diabetic patients have demonstrated spontaneous NET production even in the absence of exogenous stimuli, yet they exhibited impaired NET generation when stimulated with PMA or LPS.^463,464^ Furthermore, evidence suggests that neutrophils isolated from the blood of patients with diabetic foot ulcers exhibit increased spontaneous NET formation but impaired inducible NET generation.^465^ In vitro experiments have indicated that oxidative stress in a high-glucose microenvironment promotes NET formation,^466^ whereas contrasting results have been reported, showing impaired NET production in response to high glucose conditions in vitro.^464^ In vivo experiments present a conflicting perspective on the role of NETs in the pathogenesis of T2D. NETs are acknowledged to play pivotal roles in fostering diabetic ulcers,^181,449,467,468^ retinopathy,^469,470^ and nephropathy.^471^ Patient data suggest that severe obesity is associated with increased generation of plasmatic NETs, potentially influencing systemic inflammatory status.^472^ However, in a murine model of obesity, inhibition of PAD4 activity leads to NET reduction and attenuation of adipose tissue inflammation, albeit failing to prevent diabetes.^473^ Although the precise role of NETs in the initiation of T2D remains unclear, a clear positive correlation between NETs and the development of poorly controlled diabetes has been established.

Metabolic-dysfunction-associated steatotic liver disease (MASLD) is a burgeoning global health challenge,^474^ ranging from simple steatosis to metabolic-dysfunction-associated steatohepatitis (MASH), liver cirrhosis, and even HCC.^475,476^ Neutrophil infiltration has long been observed in human MASLD.^477^ Concurrently, plasma levels of NET markers escalate in patients with MASLD,^66^ with a gradual increase noted with disease progression.^478^ Experimental induction of steatosis in murine models correlates with excessive neutrophil infiltration in the liver.^479^ Free fatty acids (FFAs), such as linoleic acid and palmitic acid are considered to be stimulants for augmented NET formation in MASLD.^480,481^ Furthermore, cholesterol crystals, prevalent in MASLD livers,^482^ serve as potent inducer of NETs.^24^ However, inhibition of NETs through DNAse I or utilization of PAD4 knockout mice dose not impede FFA accumulation, implying that NET formation is a consequence of lipid accumulation rather than a causative factor of steatosis.^480^ MASH is a progressive form of MASLD that slowly progresses toward cirrhosis and finally leads to the development of HCC.^483,484^ Our research unveils NET formation in NASH, highlighting elevated serum levels of MPO-DNA in preoperative NASH patients.^480^ Furthermore, increased intrahepatic platelet accumulation correlates with NET formation in liver biopsies of patients with MASLD.^485^ Studies underscore the cytotoxic effects of NETs on endothelial cells,^66,486,487^ fostering a procoagulant and pro-inflammatory phenotype,^488,489^ thereby accentuating the hypercoagulable state in patients with MASH. Moreover, NETs contribute to the establishment of a protumorigenic inflammatory environment, promoting the progression of HCC in MASH.^480^ Recent study suggests that NETs play a crucial role in bridging innate and adaptive immunity by promoting Treg differentiation through metabolic reprogramming of naïve CD4^+^ T cells in MASH,^39^ thereby fostering an immunosuppressive environment for MASH-HCC initiation. In vivo blockade of NETs using PAD4^-/-^ mice or DNase I treatment attenuates the Treg activity and augments cytotoxic CD4^+^ and CD8^+^ T-cell function, thus mitigating MASH-HCC initiation and development. Collectively, NET formation emerges as a pivotal factor driving the transition from steatosis to NASH, perpetuating chronic inflammation, and fostering HCC progression by shaping an immunosuppressive microenvironment conducive to aberrant hepatocyte survival.

### Thrombosis

Thrombosis, characterized by the obstruction of normal blood flow due to blood clots in arteries or veins, precipitates various pathologies, including cerebral thrombosis, atherosclerosis, coronary thrombosis, pulmonary embolism, and deep venous thromboembolism (DVT).^490,491^ Over the past few years, the role of NETs has revolutionized our understanding of thrombosis, with studies elucidating their role in both venous and arterial thrombotic events.^308,492^ As discussed above, NETs facilitate thrombus formation by acting as a scaffold that triggers platelet activation and coagulation.^20^ Nevertheless, dysregulation or excessive NET generation precipitates pathological thrombotic processes (Fig. 5).

Recent accumulating evidence from human thrombi underscores the presence of NETs within arterial thrombi across various thrombotic pathologies, including atherosclerosis,^24,493–495^ coronary artery disease,^362,496–499^ and ischemic stroke.^500–502^ In atherosclerosis, NETs were observed in both human and murine atherosclerotic lesions,^24,354,495,503^ with cholesterol crystals identified as potential inducers of NET formation. Consequently, NETs contribute to increased expression of pro-inflammatory cytokines, fostering further immune cell recruitment to atherosclerotic plaques and exacerbating atherosclerosis.^24^ Inhibiting NET formation has shown promise in reducing atherosclerosis burden in apoliporotein-E deficient mice.^504^ Although recent histological investigations reveal abundant NETs in coronary thrombi from patients with acute myocardial infarction,^71,356,505^ the extent to which NET formation contributes to coronary thrombus formation remains unclear. Research suggests that NETs are prevalent in fresh and lytic but not organized coronary thrombi, implicating their role in thrombus propagation and stabilization, with potential degradation occurring in the older thrombi.^505^ Clinical relevance is underscored by findings linking coronary thrombus NET burden and infarct size, as well as ST-segment resolution, reflecting the potential influence of NETs on myocardial infarction outcomes.^71^ Evidence further suggests localized NET formation in acute coronary syndrome, supported by elevated NETs in the blood from lesion sites compared to other sites.^496^ Furthermore, a multicenter European study showed that neutrophils and NETs are recognized features of thrombi retrieved from patients with stent thrombosis post-percutaneous coronary intervention.^356^ Similarly, in ischemic stroke, abundant NETs are observed in occluding thrombi,^506,507^ with plasma NET markers correlating with stroke severity and outcomes.^500,508,509^ However, cerebral thrombi can originate from various sources depending on stroke etiology, with studies indicating the differential abundance of H3Cit, a marker of NETs, in cerebral thrombi of cardioembolic origin compared to other etiologies.^506^ This indicates the possibility of NETs migrating from thrombi in other locations to the brain, thereby exacerbating inflammation in thrombotic processes.

Venous thromboembolism encompasses DVT, pulmonary embolism, and clot formation in large veins.^510,511^ Animal models have demonstrated the presence of NETs within venous thrombi.^512,513^ Studies have indicated elevated levels of circulating extracellular DNA and MPO in patients with DVT compared to DVT-negative individuals.^514^ Moreover, circulating NET components have been observed to rise alongside venous thrombus development in patients.^515,516^ The identification of citrullinated histones in the inferior vena cava of DVT mice further support this conclusion.^513,517^ NET involvement in thrombosis is supported by the finding that treatment with DNase and PAD4 inhibitors blocks DVT in mice.^513,518^ Venous thrombi may exhibit a lower proportion of NETs compared to arterial thrombi, as evidenced by a study comparing patients with coronary artery thrombi and those with deep vein thrombi.^71^ NET structures are predominantly localized in the organizing regions of venous thrombi rather than the organized areas,^519^ suggesting a potential role for NETs in venous thrombus maturation rather than sustained generation. Infections can accelerate neutrophil recruitment, leading to heightened involvement of NETs in venous thrombosis. Staphylococcal infection in mice suffering from inferior vena cava ligation has shown larger thrombi containing increased neutrophils and NETs.^513^ In thrombotic events triggered by infection, such as those occurring in sepsis, the presence of NETs within lung thrombi can be observed.^110^ However, clinical data regarding NETs in venous thromboembolism are relatively limited, and the precise contribution of NETs to venous thrombosis remains to be further elucidated.

### Pregnancy-related diseases

Elevated white blood cell counts during pregnancy have been documented,^520,521^ with several studies indicating a mild neutrophilia associated with pregnancy.^520,522^ Within the context of normal pregnancy, neutrophils exhibit heightened susceptibility to activation with an augmented capacity for phagocytosis in comparison to non-pregnant women.^522,523^ Nevertheless, the precise mechanism and underlying rationale monitoring the heightened activity of peripheral blood neutrophils during pregnancy remain unknown.

Pre-eclampsia (PE), whereby activation of leukocytes such as neutrophils is enhanced, is a paramount contributor to maternal mortality on a global scale.^524,525^ Evidence suggests a detrimental role of NETs in the pathogenesis of PE.^526,527^ Histological analysis of placental tissue from patients with PE reveals the presence of NETs in close proximity with trophoblasts.^342,527,528^ An elevation of NET levels within the placental inter-villous space of PE pregnancies has also been observed.^529,530^ Concurrently, elevated levels of maternal cell-free DNA (cfDNA), a hallmark of PE^531,532^ are observed, correlating with disease severity.^533^ NETs are observed in PE as they are the main origin ofconnected to the presence of cfDNA in maternal plasma.^526,528,534^ In vitro experiments demonstrate that placenta fragments stimulate the formation of NETs by neutrophils.^534^ Meanwhile, the release of particles of syncytiotrophoblast and endothelial cell origin induce NET release.^535^ Additionally, DNA released from damaged placental cells further augments NET formation, leading to vascular endothelial cell damage through a positive feedback loop, thereby exacerbating pregnancy complications, enhancing blood coagulation, and increasing the risk of thrombotic events.^526,535^ Furthermore, placental NETs are hypothesized to provoke autoimmune reaction in PE.^527,534^ However, the precise role of NETs in initiating pathological changes remains unclear, warranting further investigations into whether NETs are triggered by placental deficiency or its consequential outcomes.

Gestational diabetes mellitus (GDM) represents a transient sate of glucose intolerance occurring during pregnancy.^536,537^ Pregnancies complicated by GDM face an elevated risk of developing PE.^538^ Notably, circulatory neutrophils in GDM cases demonstrate an exaggerated pro-NETosis phenotype, along with heightened placental infiltration evidenced by the expression of neutrophil elastase (NE).^539^ Neutrophils in GDM exhibit heightened activation, leading to spontaneous NET generation in vitro.^540^ The administration of infliximab, a clinically utilized TNF-α antagonist, notably attenuates the pro-NETotic effect of GDM sera.^540^ Additionally, degranulated neutrophil release NE, which perturbs trophoblast physiology and glucose metabolism via modulation of key signal transduction components.^539^ A study elucidates hypoadiponectinemia as a trigger for NET formation, which promotes trophoblast apoptosis through ROS-dependent mitochondrial pathway activation mediated by ERK1/2 signaling.^541^ Furthermore, induction of GDM in NETs-deficient PAD4^−/−^ mice leads to a significant increase in placental weight compared to wild-type mice,^542^ indicating a potential contribution of altered NET activity to the pathogenesis of PE in GDM.

Moreover, pregnancies frequently encounter complications such as spontaneous abortions, often associated with heightened stress or inflammatory condition.^543,544^ A study investigated a cohort of 268 women, observing a correlation between spontaneous abortions and elevated fetal cfDNA levels in maternal blood.^545^ Dysregulated LDNs have been implicated in early spontaneous abortions, exhibiting increased in vitro NET formation.^546^ Analyses revealed the presence of NETs within placental tissue from miscarried women, accompanied by elevated MPO and pentraxin 3 levels.^547^ Investigation into NETs associated with spontaneous abortion indicated heightened chorioamniotic NET levels in cases of chorioamnionitis and preterm delivery.^548^ Interestingly, PAD4^-/-^ mice displayed significantly reduced inflammatory and thrombotic response, leading to a marked decrease in pregnancy losses.^549^ The inhibition of NETs emerges as a promising therapeutic avenue for disorders associated with impaired placentation.

### Tumors

**Fig. 6:**
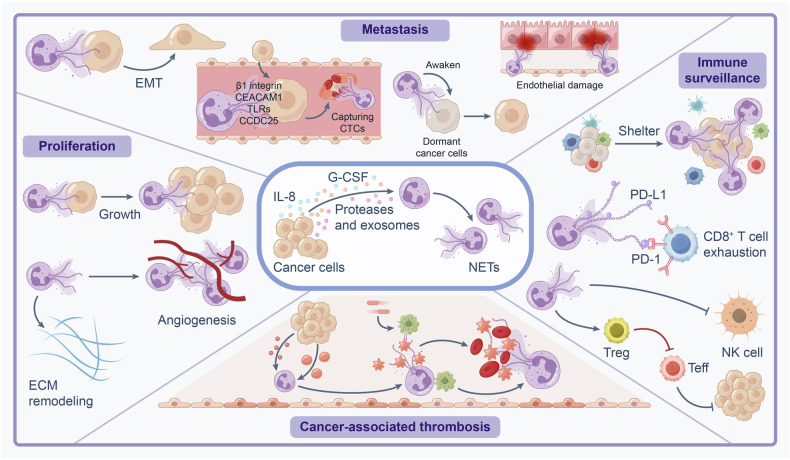
NETs in modulating cancer biology. NET components play a direct role in shaping the biology of cancer. NETs are implicated in tumor cell immunosuppression, proliferation, metastasis, and cancer-associated thrombosis. In tumor proliferation, NETs directly promote tumor growth, angionenesis, and ECM remodeling. In cancer immune surveillance, NETs may contribute to the suppressive TME by: 1. Directly affecting the killing function of NK cells and cytotoxic T cells. 2. Forming a shield to protect tumor cells from effector cells. 3. Promoting Treg activity to inhibit the function of effector cells targeting abnormal cells. For cancer metastasis, NETs capture CTCs through integrin β1, CEACAM 1, TLRs, and CCDC25. NETs also promote EMT and contribute to endothelial damage and increasing vascular permeability. Moreover, NETs can awaken dormant cancer cells at distant sites. NETs also contribute to cancer-associated thrombosis. These mechanisms are associated with the immunothrombosis function of NETs, wherein they trap platelets, red blood cells, and extracellular vesicles containing tissue factor activity, leading to vessel occlusion and promoting cancer-associated thrombosis. This figure was created by Adobe Illustrator Artwork 16.0 (Adobe Systems, USA)

NET components have been directly involved in modifying cancer biology, with emerging evidence emphasizing the protumorigenic role of NETs in various cancers.^26–28^ NETs have even been reported to favor tumor cell proliferation,^29^ metastasis,^30,31,550,551^ immunosuppression,^33,34^ angiogenesis, and cancer-associated thrombosis.^35^ Moreover, NETs can capture circulating tumor cells (CTCs) and promote their colonization.^36^ Conversely, NETs can also exhibit anti-inflammatory and anti-tumorigenic functions.^552^ They have the ability to mitigate inflammation by degrading cytokines and chemokines, as well as coordinate the resolution of sterile cancer-related inflammation.^310^ Thus, there may exist a bidirectional interplay between cancer cells and NETs (Fig. 5). Conversely, the presence of cancer cells can influence neutrophil activity, maturation, and cell fate (Fig. 6). Tumor cells have the capability to prime neutrophils to form NETs.^128^ IL-8/CXCL8 produced by cancer cells and several cancer-related stimuli (such as CXCR1/CXCR2 agonists, G-CSF, TGF-β, tumor-derived proteases, and tumor exosomes), can induce the release of NETs from both human and murine neutrophils.^201,553–556^ Besides cancer cell-derived factors, cancer-associated fibroblasts have also been identified as drivers of suicidal NETosis.^557^ Moreover, hypoxia in the TME may also induce NETs, as HIF-1 plays a critical role in NETosis and bacteria-killing activity.^558^

#### Tumor immune surveillance

Evidence indicates that NETs contribute to the creation of a suppressive inflammatory microenvironment at primary or secondary sites, thereby promoting the seeding, survival, proliferation and metastasis of primary tumor cells.^33,559,560^ CD8^+^ T cells, key effectors in the anti-cancer immune response,^561,562^ interact with NETs in the TME, as confirmed by the negative correlation between NET density in the serum of patients with cancer and CD8^+^ T cells in the TME.^560^ Furthermore, neutrophils isolated from patients undergoing resection of colorectal liver metastases were found to be predisposed to forming NETs, resulting in exhaustion and dysfunction of human CD4^+^ and CD8^+^ T cells,^33^ accompanied by increased expression of exhaustion markers PD-1, Tim-3, and LAG-3, along with diminished production of effector cytokines IL-2, IFN-y, and TNF-a.^33^ Mechanistic studies revealed that PD-L1 is embedded within the NET structure, suggesting that targeting PD-L1-containing NETs may prevent tumor growth, offering a novel strategy to enhance immune surveillance in the TME. NK cells, key cells in immune responses,^563^ are affected by NETs, as demonstrated by an in vitro study showing that NETs can inhibit NK cell migration and motility.^201^ In a TME abundant in NETs, the therapeutic efficacy of NK cells is impaired,^285^ possibly due to MMP9 in NETs contributing to NK cell dysfunction and tumor invasion.^564^ Inhibition of NETs in a murine model of HCC enhanced anti-tumor immunity mediated by NK cells.

Evidence has also shown that CXCR1 and CXCR2 agonists produced by tumor cells promote NET formation, which act as a protective shield against cytotoxicity mediated by NK cells and T cells.^201,565^ Additionally, studies have validated that NETs protect tumor cells by creating a physical barrier at the tumor/stroma interface,^566,567^ thus preventing the infiltration of CD8^+^ T cells into tumor cell areas. Moreover, NETs contribute to an immune suppressive microenvironment for tumor survival by interacting with Tregs. Our recent finding indicates that accumulated NETs can cause extensive hepatocyte damage and establish an immunosuppressive microenvironment for premalignant hepatocytes and cancer cell survival by promoting Treg activity,^39^ thereby facilitating the initiation and development of HCC.^480^ Inhibiting NETs may reduce the number and suppressive function of Treg and enhance the cytotoxicity of effector CD4^+^ and CD8^+^ T cells, thus preventing tumor progression. Moreover, inhibiting NET formation may sensitize cancer cells to immune checkpoint blockade.^568^ In summary, NETs may contribute to the suppressive TME through: 1) Directly affecting the killing function of NK cells and cyctoxic T cells. 2) Forming a shield to protect tumor cells from effector cells. 3) Promoting Treg activity to inhibit the function of effector cells killing abnormal cells. Targeting NET function may reprogram the impaired immune surveillance in the TME, thereby hindering tumor initiation and progression.

#### Tumor proliferation

Elevated levels of plasma biomarkers of NETs such as cfDNA, NE and citH3, have been observed in various cancers, including but not limited to pancreatic cancer,^512,568–570^ gastric cancer,^89,571,572^ and breast cancer.^42,83^ In most reports, NETs have been linked to a protumorigenic role in both experimental murine models and patients with cancers. NETs have been shown to induce endothelial-to-mesenchymal transition (EMT) in several type of cancers.^86,573,574^ In an experimental melanoma model, NETs accumulated in the TME and promoted cancer growth,^575^ a phenomenon also observed in HCC development.^126,480^ In a murine model of orthotropic pancreatic adenocarcinoma, NETs activated pancreatic stellate cells, promoting tumor proliferation, while inhibiting NETs reduced stromal activation and tumor growth.^576^ In vitro experiments have further confirmed that NETs promote tumor cell proliferation. Another mechanism through which NETs promote tumor growth is their pro-antigenic effects,^577^ possibly mediated by NETs-induced activation of endothelial cells via TLR-4/NF-kb signaling or upregulation of proangiogenic factors such as vascular endothelial growth factor.^577–579^ Whereas most evidence supports the tumor-promoting role of NETs, several studies have also demonstrated their protective role in tumors.^552^ Co-culture of melanoma cells with NETs resulted in decreased melanoma cell migration and viability.^580^ Additionally, experimental evidence suggests that NETs inhibit the proliferation of colon carcinoma cells.^581^ These controversial findings may reflect the dual role of NETs in the TME, which may vary depending on the disease stage.

#### Tumor metastasis

Several studies involving patients with various cancer types offer additional evidence supporting the involvement of NETs in promoting metastasis. Recent investigations have shown a correlation between NET levels and metastasis in HCC and breast cancer.^36,94^ The highest levels of NETs were found in metastatic lesions from patients with triple-negative breast cancer, a subtype characterized by aggressive tumor progression and high risk of metastatic spread.^582^ In a mice model with lung and colon cancer, tumour-induced NETs contribute to cancer cell adhesion to liver sinusoids.^583^ IL-8/CXCL8 mediates a positive loop connecting NET formation and colorectal cancer liver metastasis.^584^ NETs have also been identified as promoting factors in the metastasis of other cancer types, including but not limited to ovarian cancer,^551^ pancreatic dual adenocarcinoma (PDAC),^90^ cholangiocarcinoma,^585^ esophagogastric cancer,^89,583^ and also non-solid cancers such as diffuse large B cell lymphoma.^586^ Enhanced metastasis has been suppressed by treatments that inhibit NETs, such as PAD4 knockout or DNase I or NE inhibitor therapy.

Mechanistically, NETs have been implicated in promoting metastasis through several mechanisms: 1) Capturing CTCs. NETs with their web-like structure and adhesive properties, can ensnare CTCs, facilitating their spread in circulation and favoring the metastatic process.^66,587,588^ Integrin β1^589^ and CEACAM^590^ have been identified as crucial for this interaction. Additionally, the DNA component of NETs in the liver exhibits chemotactic properties for CTCs, interacting with the coiled-coil domain containing protein 25, a transmembrane protein expressed on CTCs.^36^ 2) Promoting EMT. NETs induce EMT, as evidenced in both murine model and patients.^86,97,573,574^ This ability to induce EMT in both normal and neoplastic epithelial cells suggests that NETs may contribute early in the process of neoplastic transformation.^31^ 3) Causing endothelial damage and increasing vascular permeability. Circulating NETs rapidly disrupt endothelial cells contacts, leading to endothelial damage and vascular leakage.^591^ NET-associated proteases, including NE, MPO, and MMPs, compromise junction integrity and promote vascular permeability.^592,593^ 4) Creating a premetastatic niche for cancer cells. NETs create an immune-suppressive niche for CTCs, particularly in the development of liver metastasis.^84,584^ NETs can also contribute to the premetastatic niche in lungs in mice with breast cancer.^594^ 5) Enhancing cancer cells’ metastatic abilities via NETs. The primary tumor can induce NET formation, with metastatic cancer cells showing an enhanced capacity to induce NETs compared to poorly metastatic tumor cells.^566^ Tumor-induced NETs increase breast cancer cell motility and promoted lung metastasis.^566^ Tumor-derived cathepsin C promotes metastasis through NET-dependent mechanisms.^83^

It is worth noting that NETs have been implicated in postoperative infection-related metastasis and occurrence. In 2016, we first proposed an enhanced metastatic role of NETs induced by surgical stress using a mouse model of hepatic I/R injury,^595^ which prevented metastasis by NET inhibition with DNase I or PAD4 inhibitors. Recently, we further demonstrated that I/R injury in the liver and the subsequent NET formation promote the formation of colon cancer metastasis in the lung.^84^ In this study, NETs were shown to have a higher propensity to bind CTCs aggregated with platelets. Additional evidence provided by a study confirmed that cecal ligation and puncture in mice contributed to NET formation, enhanced trapping of CTCs, and increased formation of liver metastasis.^66^ These findings suggest that infection-induced NETs enhance the trapping of tumor cells. LPS-induced NET formation was also shown to promote tumor metastases in a mouse model of CRC.^596^ Although surgical removal of the tumor may be curative clinically, inhibiting NETs as a preventive measure for postoperative infection and subsequent recurrence may provide clinical insights.

#### Tumor-associated thrombosis

The prothrombotic nature of NETs has been implicated in cancer-associated thrombosis,^109^ as evidenced by clinical data^76,597^ and mouse studies.^64,512,554^ NET complexes or components have been detected in coronary, cerebral and pulmonary thrombi in patients with various cancer types.^40,598^ Elevated circulating NET markers predict a higher risk of VTE in patient with cancer.^76^ Moreover, circulating NET markers are elevated in HCC-associated portal vein thrombosis^599^ and cancer-related stroke.^509^ These mechanisms are related to the immunothrombosis function of NETs, which trap platelets, red blood cells, and extracellular vesicles with tissue factor activity, occluding vessels and promoting cancer-associated thrombosis. Specifically, 1) cancer-induced platelet activation and NET release contribute to the hypercoagulable state in cancer;^600,601^ 2) tumor-derived pro-coagulant micro particles promote DVT by carrying tissue factor and adhering to thrombus-associated NETs;^602^ 3) NETs released from cancer patients increase levels of thrombin-ant thrombin complexes and enhance the ability of control plasma to generate fibrin.^41^ Administration of DNase I reduced thrombus size in mice bearing human tumors.^512,603^

#### Tumor prognosis

While the clinical significance of circulating NET molecules as cancer biomarkers remains a debate, recent evidence suggests a direct correlation between the high levels of NET markers and poor clinical outcomes in patients with cancer.^40–42^ Elevated level of H3Cit has been identified as an independent prognostic factor for short-term survival in cancer patients.^604^ In patients with colorectal cancer, elevated pre-operative circulating levels of cfDNA have been linked to persistent disease one year after resection.^605^ Additionally, in patients with metastatic colorectal cancer undergoing curative liver resection, high levels of circulating NET markers are associated with a high risk of recurrence and worse prognosis.^595,596^ Similarly, in patients with breast cancer, cfDNA correlated with tumor size, nodal involvement, and clinical stage.^606^ Serum NET levels can predict the occurrence of liver metastasis in patients with early-stage breast cancer.^36^ High NET density is correlated with lower recurrence-free survival in patients with cervical cancer,^607^ suggesting that combining NET density with the TNM staging system could improve prognostic accuracy. NETs are also reported as a novel biomarker to predict recurrence and overall survival,^608^ and they correlate with the degree of liver dysfunction in patients with HCC. In human large B cell lymphomas, intratumoral and circulating NETs correlate with worse overall survival and progression-free survival.^586^ Plasma NET markers have been documented to correlate with poor prognosis in head and neck cancer,^609,610^ gastric cancer,^611,612^ rectal cancer,^613^ renal cancer,^614^ and pancreatic cancer.^570^ Moreover, cancer cells from a primary tumor can enter a dormant state and remain clinically undetectable for extended periods (Fig. 6). NETs have been shown to awaken dormant cancer cells at distant sites,^615^ suggesting that therapies targeting the prevention of dormant cell awakening by NETs could potentially extend the survival of cancer patients.

## NET-targeting therapies

### Targeting NET formation

Multiple pathways have been identified in the formation of NETs, and have been exploited in attempts to inhibit formation in order to abrogate negative downstream effects. A majority of the work in inhibition of NET formation has been done in the pre-clinical setting, with peptidyl arginine deaminase (PAD) being the most common target of interest (Table 1). The PAD family of enzymes catalyze the citrullination of histone proteins, a key component of NET formation.^616^ Multiple prior studies have demonstrated the correlation between NET reduction and PAD inhibition, and genetic knock outs of PAD have demonstrated similar phenotypic endpoints as prohibiting NET formation. Cl-amidine has been a recently explored PAD inhibitor, used in a variety of inflammatory disease models, including lupus, diabetes, and endometritis.^458,617,618^ Shen et al. demonstrated the utility of inhibiting PAD4-mediated NET formation with Cl-amidine as a means of preventing diabetes development.^458^ In their study, Cl-amidine was administered orally at a dose of 5μg/g, resulting in a delay in onset, decreased disease incidence, and decreased type 1 diabetes-associated antibodies, which was simultaneously associated with a reduction in serum NET markers. Furthermore, these findings translated phenotypically, with inhibited pancreatic inflammation and increased regulatory T cell presence within pancreatic lymph nodes. Separately, Knight et al. demonstrated Cl-amidine could confer protective effects against specific lupus phenotypes.^617^ In their model, MRL/lpr mice, which are more prone to accelerated lupus phenotypes, were treated with subcutaneous injections of either 10 mg/kg/day of Cl-amidine, 1 mg/kg/day of BB-Cl-amidine, a more bioavailable form of Cl-amidine. PAD inhibition with these agents resulted in reduced proteinuria and immune complex deposition, as well as downregulation of type I interferon production in a murine model otherwise prone to developing severe disease.

**Table 1:** Pre-clinical studies targeting NET formation and structure

Intervention	Species	Target/Disease of Interest	Results	Reference
Pre-clinical Studies Targeting NET Formation				
Hydroxyethyl starch 130/0.4	Mouse	Platelet-neutrophil aggregate	Reduced aggregates Reduced NET formation	Rossaint et al.^621^
Secretory leukocyte protease inhibitor	Mouse	Neutrophil elastase	Reduced NET formation	Zabieglo et al.^634^
Gallic acid	Human	LPS-induced apoptosis	Reduced NET formation Reduced free radical formation Decreased apoptosis	Haute et al.^635^
Prostaglandin E2	Mouse	Cyclic AMP	NET formation inhibited	Shishikura et al.^636^
Activated protein C	Human	Mac-1 EPCR PAR3	Leukocyte binding Inhibited NETosis	Healy et al.^637^
rhThrombomodulin	Rat	Shock Induced Coagulopathy	Reduced NETosis Attenuated coagulopathy	Helms et al.^620^
Azithromycin/Chloramphenicol	Human		Reduced NET formation Azithromycin – dose dependent effect on neutrophil respiratory burst	Bystrzycka et al.^638^
Cl-amidine	Mouse	Peptidyl arginine deiminase (PAD) Lupus	Reduced NET formation Renal protection	Knight et al.^616^
Cl-amidine on polydioxanone	Rat	PAD4	Dose-dependent inhibition of NETosis	Fetz et al.^639^
Cl-amidine	Mouse	PAD Diabetes	Reduced serum PAD4 and MPO-DNA Inhibited pancreatic inflammation Decreased IA2A	Shen et al.^458^
Cl-amidine	Rat	PAD4 Endometritis	Reduced CitH3 and HMGB1	Shen et al.^618^
rhThrombomodulin	Rat	Histone-induced NET formation	Inhibition of NET formation	Shrestha et al.^640^
Thrombomodulin	Human	HMGB1	Inhibited NET formation Reduced pancreatic metastasis to liver	Kajioka et al.^97^
rhThrombomodulin	Mouse	Endotoxin induced acute kidney injury	Reduced serum H3 and CitH3 Abolished CitH3 expression in renal medulla	Harada et al.^619^
MitoQ	Mouse	Mitochondrial oxidative stress Lupus	Reduced NET formation Reduced kidney immune complex deposition Reduced serum IFN-I	Fortner et al.^641^
BMS-P5	Mouse	PAD4 Multiple Myeloma	Abrogated NET formation Slowed disease progression	Li et al.^642^
Kaempferol	Mouse	NADPH/ROS pathway Breast Cancer	Decreased citH3 expression Decreased primary breast tumor growth and lung metastasis	Zeng et al.^643^
Chloroquine	Mouse, Correlative Human serum	PAD4 Pancreatic cancer	Exclusive PAD4 inhibition Reduced serum CitH3 (dose dependent)	Ivey et al.^644^
Low dose Vitamin D	Rat	Bronchopulmonary dysplasia	NET inhibition Increased survival Attenuated developmental retardation Improved alveolarization Arrest in hyperoxia induced BPD	Chen et al.^645^
Etanercept	Mouse	TNF-alpha	Absent NET formation TNF-induced IL-6 inhibition Reduced TNF Decreased neutrophil recruiting chemokines	Sudo et al.^646^
RNase A	Mouse	Muscle Ischemia	Reduced leukocyte infiltration Reduced MPO/CitH3 Reduced M1 polarization	Lasch et al.^579^
Zinc	Human and mouse	H3 Citrullination	Inhibited NET release	Kuzmicka et al.^622^
GSK484	Mouse	PAD4 Renal I/R	Reduced lung injury Reduced NET formation Reduced inflammatory factor secretion	Du et al.^647^
Thioredoxin-albumin fusion protein (HSA_Trx)	Mouse	Oxidative stress Pollution induced lung injury	Reduced dsDNA, citH3, neutrophil elastase in bronchoalveolar fluid	Tanaka et al.^648^
Iron/Zinc chelators	Human	-	TPEN/IDA - inhibit NET release DFO – stimulates NET release DTPA – no NET effect	Kuzmicka et al.^623^
Anakinra	Human	IL-1 Receptor	Dose and time dependent inhibition of NET and cfDNA	Wadehn et al.^649^
Curosurf, Alveofact	Human	-	Dose-dependent inhibition on NET formation Lower NE, NPO, and cDNA	Schulz et al.^650^
Antithrombin + GSK484	Mouse	PAD4	Reduced postoperative adhesion formation. Prevention of tPA-inhibitor- 1 and IL-6 expression.	Sudo et al.^651^
TcpC	Mouse	PAD4	NETosis inhibited	Ou et al.^652^
Fostamatinib	Human	SYK COVID-19	Prevents COVID-19 induced NETosis	Strich et al.^653^
Chikusetsusaponin V (CKV)	Mouse	Caspase-1 HMGB-1 Liver injury	Pretreatment interfered with NET formation Interfered Caspase-1 and HMGB-1 release in APAP damaged hepatocytes	Liu et al.^654^
Tetramethylpyrazine	Rat	Ischemic injury	Reduces NET formation Alleviates hepatic I/R injury	Liu et al.^655^
PDE4 inhibitor	Mouse	Cystic fibrosis lung disease	Reduced cfDNA in BALF Reduced citrullination of airway H3	Totani et al.^656^
Ibuprofen + GS-561937	Bovine	RSV	Reduced NETs in lung tissue (day 3)	Mutua et al.^657^
Senkyunolide I	Mouse	Sepsis-induced lung injury	Reduced lung injury by BALF Decreased lung and plasma NETs	Zha et al.^658^
Disulfiram	Mouse	Gasdermin D Sepsis-induced organ failure	Reduced circulating NETs Reduced CKMB, BUN, AST Reduced gross histopathological changes	Silva et al.^323^
Disulfiram	Mouse	GSDMD Severe acute pancreatitis	Alleviated pancreatic inflammatory injury. Reduced NET expression	Ling et al.^624^
Reparixin	Mouse	CXCR1/2 Sepsis	Reduced NET formation Reduced multi-organ injury Reduced mortality	Alsabani et al.^659^
Manganese	Mouse	S. Aureus Infection	Decreased mitochondrial superoxide Decreased suicidal NETosis	Monteith et al.^660^
Itaconate (4-OI)	Mouse	LPS-induced NET release	Reduced formation by 4-OI and downstream HIF-1a inhibitor	Burczyk et al.^661^
Salvianolic Acid A	Mouse	LPS-induced lung injury	Ameliorated lung injury Reduced NETosis	Liu et al.^662^
Nanoflower – ZD-E-1	Mouse	PAD4 Lung cancer	Self assemblign carrier free drug inhibtiing PAD4 and NET formatino, improving TIME	Zhu et al.^663^
Taurine	Mouse	S. Uberis Mastitis	Inhibited NADPH oxidase Reduced NET production	Li et al.^664^
Ivermectin	Mouse	GSDMD Melanoma metastasis	Suppressed GSDMD oligomerization Reduced NET formation, Reduced ecDNA	Zhang et al.^665^
Liraglutide	Mouse	Lung and liver cancer with checkpoint inhibition	Decreased circulating MPO, NE, dsDNA. Downregulated ROS species in TME Enhanced PD-1 activity	Chen et al.^666^
(+)-Borneol	Human	Oxidative stress	Pre-treatment inhibited PMA induced NETosis Inhibited ROS burst Abrogated effects of TLR2 inhibition	Chen et al.^667^
IL-37	Mouse	Acute viral myocarditis	Improved cardiac function Inhibits inflammatory cell infiltration Inhibits NET formation	Li et al.^668^
Dihydrotanshinone I (DHT)	Mouse	TIMP1 expression Breast cancer	Blocked lung metastasis Reversed NET formation Ameliorated NET-induced metastasis Inhibited neutrophil infiltration into lung Reduced CitH3 expression in lung	Zhao et al.^669^
JBI-589	Mouse	PAD4 Rheumatoid arthritis	Decreased MPO, CitH4 Decreased clinical markers of RA	Gajendran et al.^670^
Taxifolin	Mouse	Nrf2 Lupus/APLA	Reduced in vivo NETosis Attenuated autoantibody formation Attenuated inflammatory cytokine production	Rysenga et al.^671^
Irisin	Mouse	Integrin AlphaVbeta5 Acute pancreatitis	Reduced NET formation in pancreatic necrotic tissue	Han et al.^672^
Aspirin/Ticagrelor (DAPT)	Mouse	Platelet Intrahepatic cholangiocarcinoma	Reduced micrometastasis Reduced NET induction	Yoshimoto et al.^585^
Rosavin	Mouse	Sepsis induced lung injury	Reduced BAL inflammatory mediators Decreased NET formation Decreased NET/MPO activity	Gao et al.^673^
Cyclosporine A	Mouse	Ulcerative colitis	Decreased NET formation Decreased cellular ROS	Xu et al.^440^
Resveratrol	Mouse	SIRT1 Breast cancer metastasis to lung	Suppressed NET formation Reduced serum NE and MPO-DNA Increased CD8 infiltration to lungs	Yu et al.^674^
Thymopentin	Mouse	Crohn’s disease	Ameliorated weight loss Reduced disease activity index (DAI) Increased TNF-a, IL-1b, IL-6. Decreased MPO, NE, CitH3, dsDNA Tissue CitH3 correlated with DAI and TNF-a	Cao et al.^441^
**Pre-clinical Studies Targeting NET Structure**				
Epigallocatechin-3-gallate	Mouse	Neutrophil Elastase Severe acute pancreatitis	Reduced pancreatic tissue damage Reduced systemic inflammatory response	Li et al.^88^
DNase1	Mouse	Wound healing	Improved scar appearance Improved collagen deposition Reduced fibrin concentration Reduced wound closure time Reduced NET presence	Heuer et al.^675^
DNase1	Mouse	Endometritis	Reduced MPO activity Reduced pro-inflammatory cytokine production Reduced CitH3 levels	Hao et al.^676^
DNase1	Mouse	Endometritis	Reduced MPO activity Reduced pro-inflammatory cytokine production Reduced CitH3 levels	Hao et al.^676^
rhDNase	Mouse	Breast cancer associated thrombosis	Prevented thrombus formation Long term treatment reduced OS Attenuated mortality	Varady et al.^603^
RhDNase-I	Mouse	ARDS	NETs reduced lung tissue Attenuated platelet-NET aggregate Reduced platelet activation Normalized clotting time	Jarrahi et al.^677^
DNase1	Mouse	Liver I/R injury	Protected hepatocytes and reduced inflammation	Huang et al.^375^
DNase1	Rat	Intestinal I/R injury	Reduced intestinal neutrophil infiltration Reduced histone and MPO complexes histone MPO complexes	Boettcher et al.^678^
DNase1	Rat	Intracerebral hemorrhage	Reduced ICH-induced NETs Improved tPA induced hematoma fibrinolysis Relieved cerebral edema Reduced cell death Improved functional outcome	Tan et al.^679^
DNase I	Mouse	Diabetic keratopathy	Reduced NETs on corneal epithelium. Reactivated epithelial regeneration signaling pathways Attenuated ROS accumulation Restored impaired corneal sensitivity in diabetic mice	Zhang et al.^680^
DNase	Rat	CSF block in early pneumococcal meningitis	Restored glymphatic transport Reduced brain weight	Pavan et al.^681^
DNase 1	Mouse	Thrombotic Stroke tPA Resistance	Promotes NET lysis but not tPA lysis Promotes ex-vivo platelet thrombi Recanalized occluding vessels	Pena-Martinez et al.^682^
DNase1	Mouse	Neurogenic pulmonary edema after SAH	Decreased lung water, neutrophilic infiltration, and inflammation. Reduced NETs and proinflammatory macrophage transition	Wu et al.^683^
DNase	Human	Trauma thrombin generation	Shorter lag time, shorter time to peak thrombin generation Decreased cfDNA Decreased citH3	Goswami et al.^684^
DNase1	Mouse	COVID induced multiorgan injury	Decreased detectable levels of NETs Reduced lung, heart, and kidney injuries	Veras et al.^685^
DNase1	Rat	IGA vasculitis	Reduction in serum cfDNA and MPO-DNA Decreased NET in renal, gastric, and duodenal tissues Lower renal MPO and CitH3 expression	Chen et al.^686^
DNase1	Mouse	MASH-HCC	Decreased tumor growth	Van der Windt et al.^480^
DNase 1 + AuPB/mPDA shell	Mouse	Colorectal cancer	Abolished metastatic seeding	Chen et al.^588^
DNase + PD-1	Mouse	Colorectal cancer	Improved CD8 infiltration Reversal of anti-PD-1 resistance	Zhang et al.^687^
AAV-DNase I	Mouse	Colorectal cance	Recruited CD8^+^ T cells to CRC liver metastasis Reduced the growth of liver metastasis	Xia et al.^92^
DNase1 + sivelestat	Rat	I/R injury	Reduced NET’s Attenuated muscle fibrosis Improved motor function DNase performance superior topically, sivelestat performance superior when IV	Wang et al.^688^
Statins	Mouse	DVT and Post thrombotic syndrome	Reduced stasis venous thrombus burden Reduced platelet aggregation and clot stability Reduced PAI-1, TF, MPO, NETs	Kessinger et al.^689^
RhADAMTS13	Mouse	Skin allograft	Absent NETs Lessened inflammation	Wong et al.^690^
CEACAM1 -blockade	Mouse	Metastatic colon cancer	Decrease in cell adhesion, migration, metastasis	Rayes et al.^590^
Exenatide	Mouse	Colon cancer cells	Restricted tumor growth when combined with anti PD-1 Reduced ROS production Reduced in vitro NETs	Chen et al.^626^
Physical activity	Human	Outcome after CV events	Decrease in cfDNA Increase in endogenous DNase activity	Ondracek et al.^625^
Hochuekkito	Mouse	UVB radiation	Suppressed inflammation, Inhibited ROS and H2O2 generation Reduced CitH3 and PAD4	Inaba et al.^691^

NET formation can also be targeted through blocking histone citrullination directly. Agents such as thrombomodulin have been studied in this role and applied to a broad range of disease, including sepsis-mediated injury, coagulopathy, and cancer.^97,619^ Helms et al. explored the use of recombinant human thrombomodulin in rat models of shock-induced coagulopathy, and found that administration of rhThrombomodulin not only decreased histone-induced NETosis, but attenuated the coagulopathy control rats experienced.^620^ In a model of endotoxin-mediated renal injury, Harada et al. established that intraperitoneal administration of 6 mg/kg of rTM following LPS-induced septic injury decreased citrullinated histone H3 levels in the serum and renal medulla,^619^ suggesting rTM could suppress NET production. Although this study did not connect these immunohistologic and serologic findings with a phenotypic benefit, other groups have demonstrated the phenotypic benefits of rTM. Kajioka et al. studied this in the context of pancreatic cancer,^97^ finding that thrombomodulin degraded HMGB1 with consequential inhibition of NET induction, leading to prevention of surgically-induced pancreatic metastases to liver.

In addition to these novel agents, there has been a wave of repurposing commercially available drugs to target NET formation. Hydroxyethyl starch, which no longer has utility as a colloid agent, was administered at a dose of 20 mg/kg by tail vein injection in a group of mice undergoing cecal ligation and puncture as a sepsis model by Rossaint et al. This model was found to reduce NET formation and reduce platelet-neutrophil aggregates and transmigration of neutrophils under inflammatory conditions.^621^ Zinc chelators have additionally been found to modulate NET formation through multiple studies from Kuzmicka et al.^622,623^ These in vivo and in vitro studies have demonstrated that low levels of zinc either through decreased dietary ingestion or through direct chelation led to increased NET release and enhanced neutrophil degradation, and that supplementation of zinc can inhibit histone citrullination and subsequent NET release.

While research with these agents is still in its infancy, certain drugs have already been associated with clinically relevant outcomes. Disulfiram, for example, has been found to reduce NET expression through gasdermin D inhibition, and alleviated severe inflammatory injury in acute pancreatitis.^624^ Ling et al. demonstrated in a murine model of severe acute pancreatitis induced by caerulein and LPS that treatment with either 50 mg/kg or 100 mg/kg of disulfiram led to inhibition of gasdermin D and resultant decrease in in-vivo NET formation, in turn alleviating inflammatory injury.^624^

### Targeting NET structure

Aside from prohibiting NET formation altogether, multiple preclinical studies have examined how to degrade or diminish the functionality of already formed NETs (Table 1). DNase has been the longest-studied agent, targeting the extracellular DNA component of NETs. Exogenous DNase administration has been utilized in a variety of disease states, and has consistently demonstrated reductions in measurable biomarkers, as well as associated with outcome improvements, including reversal of coagulopathies and thrombotic burdens, decreased cancer growth and metastasis, and suppression of pro-inflammatory cytokine production.

While extracellular DNA is often the target for NET degradation, there is an increasing amount of research focusing on targeting NET-associated proteins, which contribute to its functional properties. A 2020 study from Rayes et al. explored CEACAM1, a NET-associated molecule, as a therapeutic target to prevent the metastatic progression of colon adenocarcinoma. Using a murine model, they were able to identify that blocking CEACAM1 or knocking it out led to a decrease in cancer cell adhesion, migration, and metastasis.^590^ In 2023, Zhang et al. examined the effects of epigallocatechin-3-gallate (EGCG), a naturally occurring neutrophil elastase inhibitor. Through co-culturing neutrophils from peripheral blood samples from human subjects and co-culturing them with SW480 colon cancer cells and inducing NETs, treatment with varying concentrations of EGCG led to suppressed NET formation, decreased expression of STAT3 and CXCL8 in colon cancer cell-derived neutrophils, and impaired cancer cell migration and invasion.^88^

Other groups have attempted to induce endogenous endonuclease function as opposed to delivering an exogenous agent. Ondracek et al. found that endurance training led to an increase in endogenous DNase activity and a decrease in cfDNA levels, theorizing this could result in improved cardiovascular outcomes.^625^ Furthermore, some groups have opted to use agents that target downstream functions, as opposed to direct structural targeting. For example, Chen et al. examined exenatide, a glycemic control agent that had been demonstrated to downregulate ROS in prior studies, and found that as a byproduct, NET reduction was observed.^626^ After subcutaneous inoculation of MC38 colon cancer cells, 24 nmol/kg/day of exenatide, twice weekly 250 μg doses of anti PD-1 or a combination of therapy was administered. Exenatide treatment led to decreased infiltration of NETs in tumor, and decreased peripheral MPO-DNA. In vitro studies demonstrated exenatide alone decreased NET formation and release. However, combining exenatide with anti-PD-1 therapy was superior at restricting tumor growth to either agent alone, and confirmed this was related to NET interaction by demonstrating that NET degradation with 5 mg/kg DNase weakened the efficacy of the combination therapy. Generally, these preclinical studies show consensus that NET degradation or functional NET inhibition is achievable through multiple mechanisms, and results in favorable outcomes.

### Clinical trials

In the realm of human clinical trials, substantial work has been done with observational methodology, specifically post-hoc analysis of other randomized trials (Table 2). The 2022 study from Schaid et al. utilized post-hoc analysis of the COMBAT randomized control trial to evaluate proteomic markers of NETs in injured trauma patients. They found that more severely injured patients had elevated markers of Serpin B1 (a NETosis marker), and that elevation of serpinB correlated to higher levels of nonsurvival, fewer ICU-free days, and fewer ventilator-free days, supporting NETosis as a potential mediator of post-injury organ dysfunction. Additionally, Qiao et al. performed a post-hoc analysis of plasma biomarkers in patients from the CITRIS-ALI trial, examining the effects of high-dose IV vitamin C on surrogates of NET formation, cfDNA and syndecan1 in patients with sepsis-induced ARDS. The treatment arm displayed greater cfDNA reduction, and increased syndecan1 levels, suggesting amelioration of NETosis. Furthermore, an exploratory open-label randomized phase-2 sub-study of the PANAMO trial in 2022 examined the role of vilobelimab treatment and its effects on biomarkers of inflammation and coagulation. The PANAMO study evaluated whether vilibelimab, an anti-C5a antibody, improved survival in critically ill COVID patients. NET markers were measured over multiple time points, and it was found that the treatment arm had decreased rates of NET biomarkers, and suppressed IL8 secretion.

**Table 2:** Human clinical trials—observational and anti-NET interventional

Disease	Primary Outcome	Clinical significance	Reference
Human Observational Trials			
VTE in Cancer	VTE prediction in cancer patients	Elevated CitH3 associated with 13% RR increase of VTE. Elevated cfDNA associated with higher risk of VTE during first 3-6 months	Mauracher et al.^76^
VTE	VTE incidence D-dimer correlated risk model	Higher CitH3 and NE associated with VTE. Adding to D-dimer based risk model did not improve AUC	Smith et al.^692^
Pulmonary Embolism	CitH3	High endogenous thrombin potential, elevated CitH3, prolonged clot lysis time associated with 8x risk of PE-related death Enhanced NET formation associated with higher early mortality risk	Zabczyk et al.^693^
STEMI with PCI	Cardiac endpoints	CitH3 independent predictor of endpoint (MI, stroke, stent thrombosis, cardiovascular related death) [HR 3.74, *p* = 0.042]	Ferre-Vallverdu et al.^694^
Cardiac arrest	Predictive value for 28-day all-cause mortality	Serum cfDNA, citH3, MPO, NE higher in all arrest patients, and significantly higher in nonsurvivor group. cfDNA, CitH3, nucelosomes on first day after ROSC independent predictors of primary outcome	Li et al.^628^
Retinal vein occlusion	Biomarker and disease incidence	Plasma cfDNA, MPO-DNA, citH3 increased in RVO cases. Associated with thrombus formation	Wan et al.^695^
ACS/acute ischemic stroke	ACS/AIS Risk	dsDNA concentrations higher in ACS/AIS ACS risk – TnI, dsDNA concentration AIS – dsDNA concentration	Lim et al.^508^
Acute Liver Failure	Transplant-specific survival	cfDNA 7.1× higher in ALF MPO-DNA 2.5× higher in ALF cfDNA higher in severe disease MPO-DNA 30% higher in ALF patients who died or required urgent transplant Positive tissue NETs in 12/18 patient specimens	Meijenfeldt et al.^334^
AMI	1-year MACE	Platelet + soluble p-selecting + all NET markers strongest predictor of 1-year MACE [OR 1.94, 95%CI 1.16-3.25]	Hally et al.^696^
Ulcerative Colitis	Disease prognosis prediction	PAD4 expression associated with increasing histopathologic grade (*p* = 0.001), anatomical disease extent (*p* = 0.038), lack of therapeutic response (*p* = 0.046), subjection to radical surgery (*p* = 0.046)	El Hafez et al.^697^
Antiphospholipid syndrome	Association with thrombosis	Higher levels of circulating MPO-DNA and PAD4 expression. Higher expression in patients with recurrent thrombosis than incident or control (43.8% higher MPO-DNA, 2x higher RNA expression)	Mazetto et al.^698^
Diabetic Foot Ulcer (DFU)	Amputation probability	Serum NET levels higher in DFU group. NET amputation probability [HR 0.19, *p* < 0.01]	Ibrahim et al.^699^
DFU	Impaired wound healing	NET specific markers higher in DFU patients than in without. Tissue elastase increased in wounds with infections and delayed healing. Significantly lower healing rates and higher amputation rates in highest quartile of CitH3	Yang et al.^651^
Lupus Nephritis	Complete remission Progression to renal impairment at 24 months	Higher NET remnants in SLE Higher NET levels with active lupus nephritis compared to SLE without nephritis (Elastase *p* = 0.03, HMGB1-DNA *p* = 002) Higher NET remants in proliferative nephritis (Elastase *p* < 0.0001, HMGB1-DNA *p* = 0.0003) Higher NETs with reduced odds of complete remission [Elastase OR 2.34, *p* = 0.0007, HMGB1 OR 2.61, *p* = 0.006 Higher NETs with increased risk of progression to severe renal impairment (Elastase OR 2.84 *p* = 0.006, HMGB1 OR 2.04, *p* = 0.02)	Whittall-Garcia et al.^700^
COVID-19	Biomarker for prognostication	NET markers elevated in COVID-19 Associated with respiratory support requirement and short-term mortality Correlated with WBC, inflammatory cytokines, CRP, and markers of coagulation/fibrinolysis Contribute to immunothrombosis	Ng et al.^701^
Streptococcal Bacteremia	Cardiovascular morbidity and mortality	Higher MPO-DNA in bacteremic Higher MPO-DNA in abscess prone Strep groups (*p* = 0.02) Combined WBC counts + MPO-DNA to predict all cause 30d mortality with commensal strep BSI—lowest among patients with neither high MPO-DNA nor abnormal WBC (*p* = 0.058) This group has favorable composite outcome of MACE and all-cause mortality (*p* = 0.026)	Kuo et al.^702^
COVID-19	Association with MIS-C and CLL (Chilblain-like lesions)	Decreased NET degradation No NET elevation with asymptomatic infection Decreased NET levels with Omicron infection compared to other strains	Carmona-Rivera et al.^703^
Pleural Effusion	Diagnosis and prognostication	Highest NET marker concentration with parapneumonic effusion CitH3 (*R* = 0.66) and eDNA (*R* = 0.73) correlated with LDH (*p* < 0.001)	Twaddell et al.^704^
Deep surgical site infection	NET index predicting DSSI occurrence after laparotomy	Higher NET formation index (NFI) in DSSI group (*p* < 0.01) NFI positively correlated with APACHE II (*R* = 0.269, *p* < 0.01) and SOFA score (*R* = 0.258, *p* = 0.013) Higher risk of DSSI with NFI score NFI AUC 0.912 compared to CRP (0.748) and PCT (0.731)	Duan et al.^705^
Locally Advanced Rectal Cancer	Prognosis and predictive response to Neoadjuvant Therapy (RFS, CR, NCR)	High tissue NET density predicted poor post-operative survival NETs independent prognostic factor for RFS Low NET-density LARC had increased CD8 infiltration High NET density associated with EMT. High NET density associated with reduced likelihood of complete/near complete response	Zhong et al.^635^
Colon Adenocarcinoma	Predicting Response to Immunotherapy	NET risk score upregulated in patient samples Levels correlated with tumor clinicopathological and immune traits MPO linked to malignancy and poor clinical outcome.	Feng et al.^706^
Breast Cancer	Survival prognosis, treatment response	NET-related lncRNA risk scores Low risk groups had improved OS High risk groups enriched in immune-related functions and higher TMB Response to chemo/immunotherapy related with expression of NET related lncRNA (*p* < 0.001)	Jiang et al.^707^
High-grade serous ovarian cancer (HGSOC)	Biomarker role in disease diagnosis and management	Higher concentration of cfDNA, citH3, and calprotectin in plasma and peritoneal fluid. Neoadjuvant treatment reduced NET biomarkers in plasma, less so in peritoneal fluid	Tomas-Perez et al.^708^
Gastric Cancer	Prediction for immune cell infiltration	Low NET score linked to higher MSI-H, mutation load, immune activity. CSC index and chemotherapeutic treatment sensitivity connected to NET score.	Li et al.^709^
Gastric Cancer	OS Prediction and TME Identification	OS longer in low-risk group (*p* = 0.005) Differences in immune infiltration across groups. NE DNA independent factor affecting OS prognosis (*P* = 0.006)	Qu et al.^612^
Glioblastoma Multiforme	1-3 year OS prediction	NET signature to form risk groups High risk group more sensitive to treatment biclutamide, gefitinib, dasatinib Low risk group poor response to immunotherapy	Sun et al.^710^
Pancreatic neuroendocrine tumor	Prediction of post-operative recurrence	Positive expression of tumor NETs with worse RFS (*p* < 0.05) Independent prognostic factor for RFS (*p* < 0.05)	Xu et al.^107^
Non-small cell lung cancer	OS prediction	12-NETs lncRNA signature to develop risk score High risk group with significantly shorter OS (*p* < 0.0001) Risk score is independent predictive factor of OS [HR > 1, *p* < 0.001] NSCLC cell lines have higher levels of three adverse prognostic NET related lncRNA than normal lung cells	Fang et al.^711^
Head and Neck Squamous Cell Carcinoma	Prediction of 3 and 5 year clinical outcomes and immunotherapy response	6 NET-related genes to construct high vs low risk model Higher OS in low risk (*p* < 0.001) Higher TMB in high-risk model (*p* = 0.017) TMB positively correlated with risk score (*R* = 0.11, *p* = 0.019) Immune therapy more beneficial for low-risk patients (*p* < 0.001) Response to anticancer drugs closely correlated with expression of NET related genes (*p* < 0.001)	Chen et al.^610^
Gastric Adenocarcinoma	Diagnostic and prognostic predictive value	NET markers had better diagnostic value than CEA, CA19-9 High level of NETs correlated with lymph node metastases Blood NET markers inversely correlated with short-term efficacy of first-line treatment Negative HER2 status associated with higher baseline NETs and worse PFS	Zhang et al.^611^
Breast Cancer	Association with clinical stages	Higher levels of NE-DNA complexes in regional and distant stages compared to local disease NETs increase in proportion to disease stage	Rivera-Franco et al.^712^
Head and Neck squamous cell carcinoma	NET-related gene signature prognostic score	Seven NET-related genes to create score signature Score highly correlated with clinicopathologic and immune traits NIFK upregulated in HNSCC pateint samples NIFK required for HNSCC cell proliferation and metastasis	Li et al.^713^
Pancreatectomy	Surgically induced NET formation	CfDNA and CitH3 elevated after pancreatic resection Increased NET-inducing cytokines post-op Reduced NETs with robotic approach Increased NETs in with pancreatic leak	Ivey et al.^714^
Generalized malignancy	Peripheral blood biomarker in diagnosis and disease progression	CitH3 and cfDNA distinguishes healthy control and tumor CitH3/cfDNA increased with clinical stage Correlation between cfDNA and systemic inflammation related parameters in tumor patients Did not predict VTE in short-term	Wang et al.^715^
Clear cell renal cell carcinoma	NET pathway association with clinicopathologic features, prognosis, prediction of therapeutic benefit	NET clusters A – metabolic pathways, better survival outcome Cluster C – immune pathways, higher immune score, poorer prognosis Higher NET scores associated with immune cell infiltration, targeted drug response, immunotherapy benefits	Teng et al.^716^
Clear cell renal cell carcinoma	Validation of molecular subtype and survival prognosis	Six NET-related gene signature Good performance in predicting OS of ccRCC Signature significantly correlated with pTMN, immune infiltration, TMB, microsatellite instability, drug sensitivity	Quan et al.^717^
Breast Cancer	Prediction/prognosis and immunotherapy response	Risk signature model High risk score associated with poor immunotherapy response and adverse clinical outcomes	Zhao et al.^718^
AIS/AMI	NET composition and association with clinical outcome	NETs present in all patients with AIS, and 20.8% patients with AMI. Abundance of NET in thrombi associated with poor outcome score in AIS, and reduced EF in AMI	Novotny et al.^719^
Esophageal Cancer	OS	Leukocytosis associated with decreased OS and DFS. Leukocytosis resulted in higher intratumoral NET infiltration (*p* < 0.001) Higher levels of NET infiltration associated with worse OS and DFS (*p* < 0.001)	Zhang et al.^720^
Coronary Artery Disease	Adverse clinical outcomes (unstable angina, stroke, MI, death)	NET markers weakly intercorrelated (*R* = 0.103, *p* < 0.001) Highest quartiles of dsDNA had weakly but significantly elevated hypercoagulability markers (*p* < 0.001) Higher dsDNA in groups experiencing clinical endpoint (*p* 0.019) Upper 3 quartiles of NETS had OR 2.01 for endpoint (*p* 0.019)	Langseth et al.^362^
Community-Acquired Pneumonia	Primary – time to clinical stability Secondary length of stay, mortality	Serum NETs associated with 3.8× increased OR of 30-day mortality Elevated serum NETs associated with higher risk for clinical instability, prolonged length of stay and 30-day mortality	Ebrahimi et al.^721^
Appendicitis	Prediction of incidence and outcome	CfDNA (AUC 0.87) and CtiH3 (AUC 0.88) demonstrated excellent predictive power for appendicitis CitH3 able to distinguish noncomplicated from complicated appendicitis and predict patient outcomes, compared to WBC and CRP	Boettcher et al.^627^
Primar hepatic malignancy	RFS and OS	High pre-surgery serum NET associated with shorter RFS/OS RFS: HCC - HR 2.9, CC – HR 3.22 High CitH3 level also predicted shorter RFS/OS	Kaltenmeier et al.^608^
Pancreatic Ductal Adenocarcinoma	PFS, Disease-specific survival	Positive NET expression exhibited poorer PFS and DSS NET formation is independent prognostic predictor of DSS PDAC with negative NET staining more likely to benefit from ACT	Chen et al.^722^
Surgery	NET formation between mild and severe surgical trauma	Decreased NETosis after severe surgical trauma Suggesting inducibility of NETs after surgical trauma may be compromised	Huang et al.^723^
Age	NET production and activity	Greater NET production in elderly (>65) than adult (20-50) adults. NETs produced in elderly reduced bactericidal capacity. Higher NET size in elderly (size of extruded DNA threads)	Sabbatini et al.^724^
Exercise (HIIT)	NET production	Baseline induction of NETosis greater in older men (*p* < 0.05) HIIT reduced induction of NETosis in older men	Vidal-Seguel et al.^725^
Human Interventional Trials			
COVID-19 ARDS	Dornase alfa/MPO-DNA complex	Reduced BALF MPO DNA Improved PF ratio Improved static lung compliance In short term	Holliday et al.^340^
COVID-19	RhDNase-1 with nanoparticulate	Reduced cfDNA	Lee et al.^630^
COVID-19	RhDNase	Decreased NETs in sputum Associated wtih recovery and improved oxygenation	Fisher et al.^726^
Pancreatic cancer	Lidocaine/Circulating NETs	No improvement is OS or DFS	Zhang et al.^633^
Pre-diabetes	Metformin/Net components elastase, proteinase-3, histones, dsDNA	Reduced NET components (elastase, proteinase-3, histones, dsDNA). Better than with other glycemic agents	Menegazzo et al.^727^
Breast cancer	IV lidocaine/MPO, CitH3, VEGF	Decreased post-op expression of NETosis	Galos et al.^632^
ARF after Trauma	Inhaled Dornase Alfa	Enrolling Incidence of moderate to severe ARDS in ventilated trauma patients in ICU	NCT03368092
ACS after PCI	Colchicine	Suppresses NET formation by storing cytoskeletal dynamics	Vaidya et al.^728^
Breast Cancer	Tamoxifen/Serum NETs, drug resistance, cancer metastasis, comorbidities	Currently enrolling	NCT05056857
Lung cancer	Perioperative lidocaine/dexmedetomidine	Reduced serum MPO Reduced MMP-3	Ren et al.^729^

Observational work is not limited to post-hoc analysis of existing studies. Multiple studies have utilized serum NET biomarkers to form prognostication and prediction models for outcomes across a variety of pathologic states. Boettcher et al utilized cfDNA and CitH3 levels as predictive markers for appendicitis in adult populations, which demonstrated superior performance compared to standard-of-care white blood cell count and c-reactive protein levels.^627^ Li et al. examined serum NET markers after cardiac arrest, identifying that cfDNA and CitH3 were independent predictors of 28-day all-cause mortality.^628^ Yang et al. found that higher serum NET-specific markers, particularly CitH3, were predictive for wound healing impairment in diabetic foot ulcers and future amputation.^629^

Currently, available interventional trials are limited, and the majority use DNase analogs as the intervention of interest. Dornase alpha, an agent known to directly degrade the extracellular DNA in NETs, was tested in a 2021 nonrandomized trial of patients with ARDS secondary to COVID-19. Inhaled administration led to reduced bronchoalveolar lavage fluid MPO-DNA complexes, improved PF ratio, and improved static lung compliance, suggesting that degradation of NETs can be beneficial in this population. However, results were not sustained at 14 days, suggesting the benefit may be short-lived.^340^

Additionally, existing agents have been studied after modifications with attempts to improve drug delivery and subsequent outcomes. In 2020, a recombinant DNase1 coated with a polymer nanoparticulate was administered in COVID-19 patients to explore whether this would improve delivery and mediate neutrophil-mediated activity. Findings suggested that this nanoparticulate coating led to reduced cfDNA levels and neutrophil activation, and may be used as a therapeutic modification.^630^

Interventional trials have also taken advantage of other existing and commercially available agents, repurposing them to target NETs. A 2018 single-arm phase 2a proof of concept study examined the effect of the combinatorial rituximab and belimumab, an antibody that leads to sustained inhibition of B cell activation, to address whether autoantibodies were related to excessive NET formation. The combination therapy administered resulted in reduced NETs in patients with systemic lupus erythematosus. It had been previously demonstrated that SLE impairs NET degradation, and those NETs propagate the inflammatory response through immune complex deposition.^631^

Another agent explored in interventional trials is intravenous lidocaine, particularly in the setting of improving disease-specific outcomes after oncologic surgery. In 2020, intraoperative IV lidocaine use was explored in breast cancer surgery and associated with decreased expression of NET markers post-operatively. While this study did not directly evaluate outcomes, the study authors set a future goal of evaluating if utilizing IV lidocaine in curative intent surgery may reduce recurrence.^632^ Shortly thereafter, a multicenter randomized controlled trial in 2022 evaluated intravenous intraoperative lidocaine during pancreatectomy for malignancy. Lidocaine in this setting transiently lowered circulating NETs, however there was no difference in intra-tumoral NETs, and did not improve overall or disease-free survival.^633^

## Conclusion and outstanding questions

In recent years, the growing understanding of NETs as pivotal players in both physiological defense mechanisms and pathological processes underscores their significance in human health and disease. NETs act as a double-edged sword, offering fundamental antimicrobial defense while also contributing to tissue damage and inflammation in various diseases. The intricate interplay between NETs and the immune system, coagulation pathways, and tissue remodeling processes emphasizes their multifaceted functions. However, it is worth noting that their immune-regulatory characteristics remain largely unknown, which could be beneficial in immune defense. Several factors, including the microenvironment of the disease sites and various stimuli, determine whether NETs are beneficial or detrimental in certain conditions.

The investigation into the molecular, cellular, and biophysical mechanisms governing NET formation in physiological or pathological processes is at an early stage. Various extracellular and intracellular microbes stimulate neutrophils to initiate NETs through suicidal and vital NETosis. Current research predominantly focuses on determining the factors that induce NET formation, yet show limited elucidation of their underlying cellular mechanisms. It remains uncertain whether NET formation varies between physiological and pathological conditions, such as during immunomodulatory or antimicrobial progress, autoimmune disorders, or cancer. Additionally, there is insufficient understanding of potential variations in NET components across different contexts. The functional role of NETs depends on variations in their composition and structure. Given that NETosis follows a defined sequence of events, understanding molecules inhibiting NET formation will enhance our comprehension of the fundamental mechanisms underlying NET formation and identify new targets for modulating NETs in diseases.

The spectrum of diseases associated with NETs is gradually broadening, encompassing inflammatory disorders, thrombosis, and cancer. In autoimmune diseases, NETs, serving as potential sources of autoantigens and immune-cell activators, could significantly contribute to autoimmunity development and the break of immune tolerance. Further investigations to identify auto antigenic components in NETs structure are crucial for designing new therapies for autoimmune disease therapies. The immunomodulatory properties of NETs might be necessary for enabling an appropriate inflammatory response or for limiting inflammation and maintaining homeostasis, which necessitates further investigations. Moreover, understanding their impact on other immune cells involved in both adaptive and innate immune responses will be pivotal for future research.

Despite numerous studies identifying NETs as having tumor-promoting effects, some studies have demonstrated tumor-inhibiting effects, especially in early-stage cancer or metastasis. Generally, elevated NET levels are associated with poor outcomes in various cancers, suggesting their potential clinical utility as biomarkers. A deeper comprehension of the interplay among NETs, cancer cells, and immune responses in the TME can enhance our understanding of cancer immunotherapy resistance. Moreover, the role of NETs in immune surveillance has not been sufficiently evaluated. It is likely that NETs in blood vessels versus tissues have different consequences, indicating diverse roles for NETs depending on their location.

Existing clinical and basic research highlights the importance of developing novel therapeutics targeting both the process of NET formation and the NET structures. Future research should focus on designing interventions tailored to the specific characteristics and stages of different diseases. For instance, in the early stages of infectious diseases, it is crucial to enhance the function of NETs to eradicate pathogens. Conversely, for sterile inflammation and most advanced-stage cancers, inhibiting the formation of NETs is more advantageous. When considering NET inhibition, it is more promising to focus on regulating NET formation rather than eliminating already formed NETs. This objective can be achieved by identifying and targeting the factors implicated in the pathways initiating NET formation. Given the presence of NETs in multiple organs of the human body, they hold potential as significant modulators of both health and disease states. The dynamic regulation of NET levels in the body to sustain homeostasis presents an exciting research avenue. Although researchers have already integrated NETs into various clinical trials, the primary remaining objective in the field is to translate NET-targeted therapies into clinical practice.
